# Heterometallic Zn^II^–Ln^III^–Zn^II^ Schiff Base Complexes with Linear or Bent Conformation—Synthesis, Crystal Structures, Luminescent and Magnetic Characterization

**DOI:** 10.3390/molecules23071761

**Published:** 2018-07-18

**Authors:** Barbara Miroslaw, Beata Cristóvão, Zbigniew Hnatejko

**Affiliations:** 1 Department of Crystallography, Faculty of Chemistry, Maria Curie-Sklodowska University, 20-031 Lublin, Poland; 2 Department of General and Coordination Chemistry, Faculty of Chemistry, Maria Curie-Sklodowska University, 20-031 Lublin, Poland; beata.cristovao@poczta.umcs.lublin.pl; 3 Department of Rare Earths, Faculty of Chemistry, Adam Mickiewicz University, 61-614 Poznań, Poland; zbychuh@amu.edu.pl

**Keywords:** 3d-4f complexes, luminescence, chiral coordination core, Schiff base polynuclear coordination compounds, linear conformation

## Abstract

A series of racemic, heteronuclear complexes [Zn_2_Nd(ac)_2_(HL)_2_]NO_3_·3H_2_O (**1**), [Zn_2_Sm(ac)_2_(HL)_2_]NO_3_·3CH_3_OH·0.3H_2_O (**2**), [Zn_2_Ln(ac)_2_(HL)_2_]NO_3_·5.33H_2_O (**3**–**5**) (where **HL** is the dideprotonated form of *N*,*N*′-bis(5-bromo-3-methoxysalicylidene)-1,3-diamino-2-propanol, ac = acetate ion, and Ln = Eu (**3**), Tb (**4**), Dy (**5**), respectively) with an achiral multisite coordination Schiff base ligand (**H_3_L**) were synthesized and characterized. The X-ray crystallography revealed that the chirality in complexes is centered at lanthanide(III) ions due to two vicinally located *μ*-acetato-bridging ligands. The presented crystals have isoskeletal coordination units but they crystallize in monoclinic (**1**, **2**) or trigonal crystal systems (**3**–**5**) with slightly different conformation. In **1** and **2** the Zn^II^–Ln^III^–Zn^II^ coordination core is linear, whereas in isostructural crystals **3**–**5** the chiral coordination cores are bent and lie on a two-fold axis. The complexes **1**, **3**–**5** show a blue emission attributed to the emission of the ligand. For Zn^II^_2_Sm^III^ complex (**2**) the characteristic emission bands of f-f* transitions were observed. The magnetic properties for compounds **1**, **4** and **5** are characteristic for the paramagnetism of the corresponding lanthanide(III) ions.

## 1. Introduction

The synthesis and characterization of 3d-4f polynuclear coordination compounds are current and intensively developing field of science. The studies cover diverse and fascinating structural characteristics as well as potential application such as molecular magnets, catalysts, optical devices, luminescent probes in biology or bioactive agents [1,2,3,4,5,6,7,8,9]. Schiff base ligands constitute a convenient template for designing and synthesis of new heteronuclear 3d-4f coordination compounds. But there is still a need to study the factors that influence the coordination architecture starting from coordination modes of the ligand, through the preferential coordination number of the metal ions, ending with the presence of solvent molecules in the inner coordination sphere, which is crucial for preventing from quenching of luminescent properties due to the nonradiative energy loss processes.

**Scheme 1:**
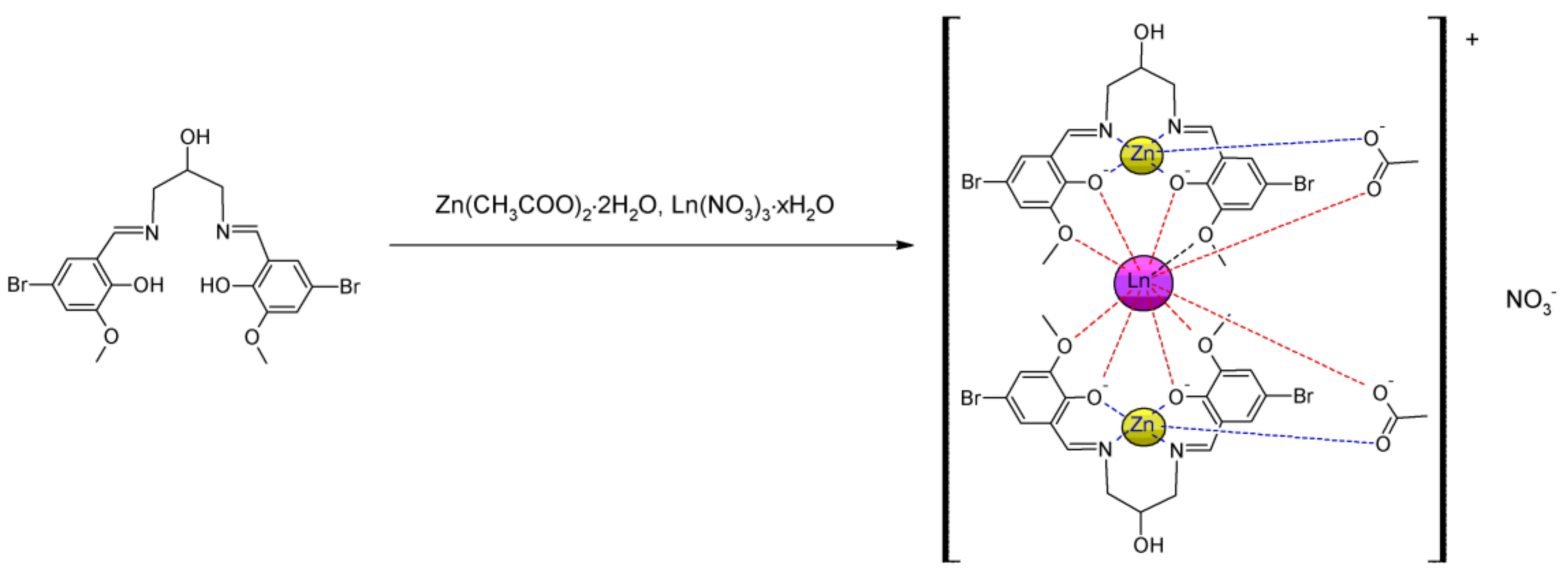
Scheme of synthesis of trinuclear coordination compounds Zn^II^–Ln^III^–Zn^II^ (Ln = Nd (**1**), Sm (**2**), Eu (**3**), Tb (**4**), Dy (**5**)) of *N*,*N*′-bis(5-bromo-3-methoxysalicylidene)-1,3-diamino-2-propanol (**H_3_L**).

We report here the heterotrinuclear coordination compounds Zn^II^–Ln^III^–Zn^II^ of *N,N*′-bis(5-bromo-3-methoxysalicylidene)-1,3-diamino-2-propanol (**H_3_L**) (Scheme 1).

There is only one report in the Cambridge Structural Database (ver. 5.39 with updates) [10] of a crystal structure of coordination compounds of this ligand. These are two homotetranuclear coordination compounds of Co^II^ and Ni^II^ and a homotrinuclear complex of Cd^II^ [11]. The trinuclear complex of Cd^II^ is reported to have photoluminescent properties with luminescence decay of the order of 10^2^ ns. The presence of Br atom in the ligand structure (heavy halogen effect) often causes a decrease of luminescence intensity and a shift in the ligand emission position [11,12], but sometimes it may be beneficial for the luminescent properties as well as for the intermolecular interactions, which can stabilize the crystal structure [13,14,15]. The ligand molecules with 1,3-diamino-2-propanol bridge may undergo double or triple deprotonation forming N_2_O_2_ or N_2_O_3_ coordination pockets (Table S1). The doubly deprotonated ligands form classic mono- up to tetranuclear coordination compounds [11,16,17,18,19,20,21,22,23,24,25,26,27,28,29]. But only the triple deprotonation enables the higher nuclearity of complexes: to hexa- [22,30] and heptanuclear units [31] with the use of additional anions such as nitrate or acetate.

The control over the diversity of coordination compounds is not a trivial problem. In the presented here complexes **1**–**5** an issue of chirality arises. Around the lanthanide(III) cation is formed a stereogenic center because of the vicinal arrangement of two *μ*-acetato-bridged ligands. The metal centered chirality may provide compounds with very interesting features applicable in modern technology. We discuss here the aspect of the linearity of the Zn^II^–Ln^III^–Zn^II^ metal core in the molecular and crystal structure as well as the luminescent properties registered in a solution and in a solid state of compounds **1**–**5** and the magnetic properties of **1**, **4** and **5**.

## 2. Results

### 2.1. Crystal Structure

**Figure 1:**
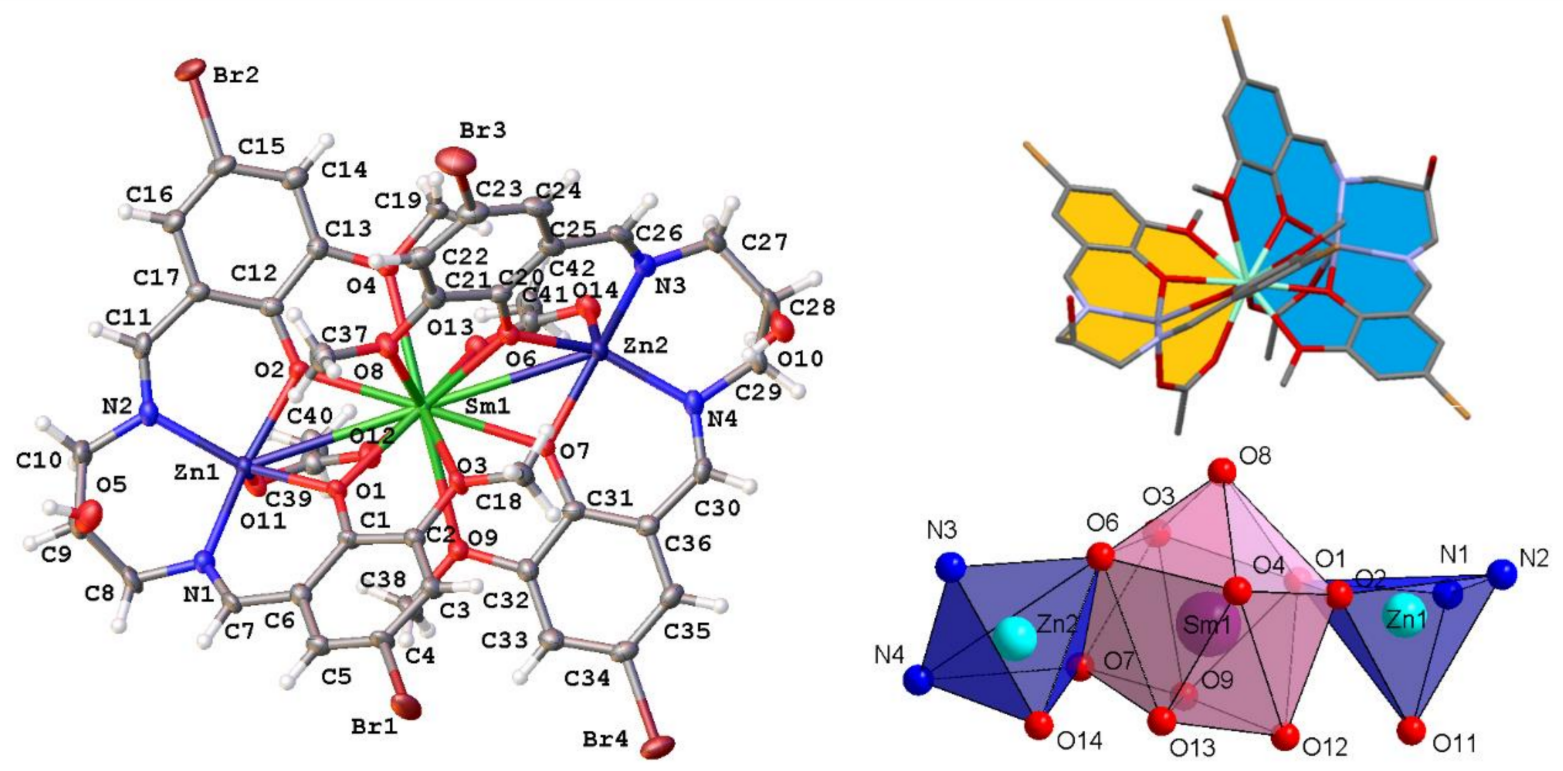
Molecular structure of **2** (Zn^II^–Sm^III^–Zn^II^) with a propeller molecular shape and coordination polyhedra around the Zn^II^ and Sm^III^ ions. The outer coordination sphere molecules were omitted for clarity.

**Figure 2:**
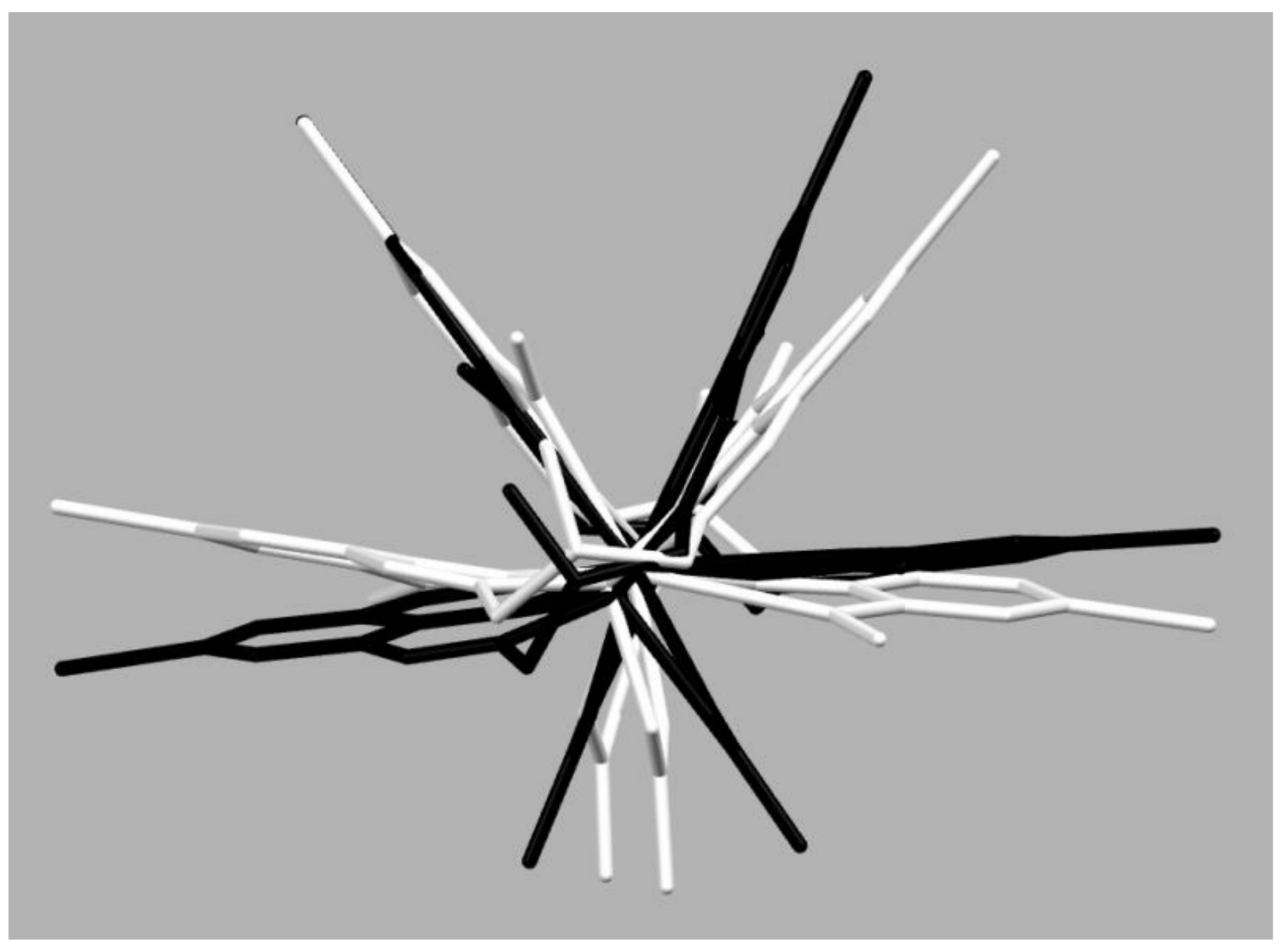
Overlay of coordination units in monoclinic structure of **2** (Zn^II^–Sm^III^–Zn^II^) (white) and in trigonal structure of **3** (Zn^II^–Eu^III^–Zn^II^) (black)—view along the Zn^II^–Ln^III^–Zn^II^ line.

The crystallographic details of structures **1**–**5** and the selected molecular geometric data are given in Table 1 and Table S2 (in Supporting Materials). The complexes **1** and **2** crystallize in the monoclinic space group *P*2_1_/*n* (**1**, **2**) but with different outer coordination spheres with general formulae [Zn_2_Nd(ac)_2_(HL)_2_]NO_3_·3H_2_O (**1**) and [Zn_2_Sm(ac)_2_(HL)_2_]NO_3_·3CH_3_OH·0.3H_2_O (**2**). The trigonal crystals **3**–**5** (space group *R*-3*c*) with formula [Zn_2_Ln(ac)_2_(HL)_2_]NO_3_·5.33H_2_O are isostructural (Ln = Eu (**3**), Tb (**4**), Dy (**5**)). The coordination units in complexes **1**–**5** are isoskeletal but have different conformations and different intermolecular interactions. The syntheses gave as a result cationic trinuclear Zn_2_Ln coordination cores with a propeller like arrangement of the ligands (Figure 1) but different from the one observed in the homotrinuclear complex of Cd^II^ [11]. The Zn^II^ ion occupies the N_2_O_2_ cavity of the doubly deprotonated Schiff base ligand **HL^2−^** from which the metal cation is deviated by ca. 0.5 Å (parameter Δ in Table 1). The square pyramidal geometry of Zn^II^ is supplemented by the acetate O atom at the apical position. The intermetallic distances Zn^II^–Ln^III^ within the core are in the ranges of 3.366(2)–3.556(1) Å (Table 1). The Ln^III^ ion is ten-coordinated and has deformed bicapped anticube polyhedron (Figure 1). The dihedral angle *σ* between ZnO_(phenoxo)2_ and LnO_(phenoxo)2_ planes and the valence angles Zn–O–Ln do not change much from monoclinic to trigonal structures (Table 1). Although, the ligand molecules are achiral, **1**–**5** form chiral coordination units crystallizing as racemic compounds in centrosymmetric space groups. The two acetate ions form bridges between Zn^II^ and Ln^III^ ions. Their *cisoid* spatial arrangement results in the formation of complexes with the stereogenic center located at the lanthanide(III) ion. The torsion angle ω between the vectors along the attitudes of the tetragonal pyramids O11–Zn1–Zn2–O14 in **1** and **2** is ca. 35° and the analogous torsion O6–Zn1–Zn1*^i^*–O6*^i^* (symmetry code: *^i^* 1/3+x-y, 2/3-y, 1/6-z) in structures **3**–**5** is ca. 62° (Table 1, Figure 2). The torsion between two N_2_O_2_ mean planes (*ε* in Table 1) is of ca. 42° for **1**, **2** and 62° for **3**–**5**.

**Figure 3:**
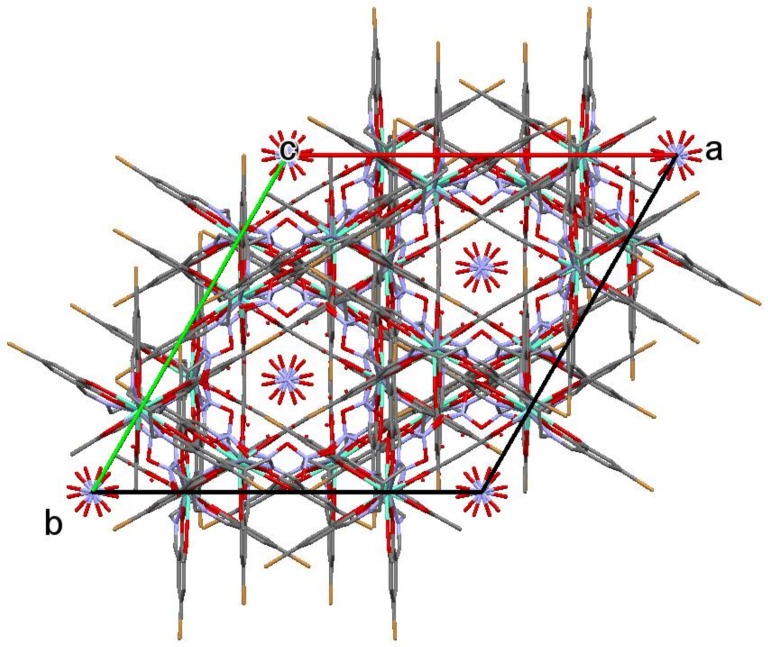
Crystal packing in **3** (Zn^II^–Eu^III^–Zn^II^)—view along the *c* axis.

Usually, the analogous angle in 3d-4f heterotrinuclear salen type Schiff base complexes bridged by *μ*-acetato anions is in the range of 14–49° [32,33,34,35,36,37,38,39,40] which means they have chiral cores, or 180° when the structures are not chiral [41,42]. The coordination units in trigonal structures **3**–**5** have a unique structural conformation for such voluminous molecules. The complexes viewed along the Zn^II^–Ln^III^–Zn^II^ axis resembles a pseudohexagonal star (Figure 2) with planes formed by 4-bromo-2-(iminomethyl)-6-methoxyphenol molecular fragments and lying opposite to them acetate ions repeated by ca. 60°. Very important difference in geometry of monoclinic and trigonal structures is the arrangement of metal ions Zn^II^–Ln^III^–Zn^II^ in the coordination cores, which is linear in complexes **1** and **2** and bent one in structures of **3**–**5** with the valence angle *φ* being of about 173° (Table 1). The hydrophilic pockets near the protonated hydroxyl groups at the propyl bridges are occupied: one by NO_3_^−^ anion and the other one by a solvent molecule (water in **1** or by disordered water and ethanol molecules in **2**) (Figures S1 and S2). In structures **3**–**5** the both symmetrically equivalent pockets are filled with substitutional disordered nitrate ions and water molecules (coordination units lie on a twofold axis) (Figure S3). Additionally, in trigonal crystals the nitrate ions are located at the three-fold rotoinversion axis in the tunnels running along the *c* crystallographic direction (Figure 3). There are no π stacks in the analyzed crystal structures.

### 2.2. Luminescent Properties

**Figure 4:**
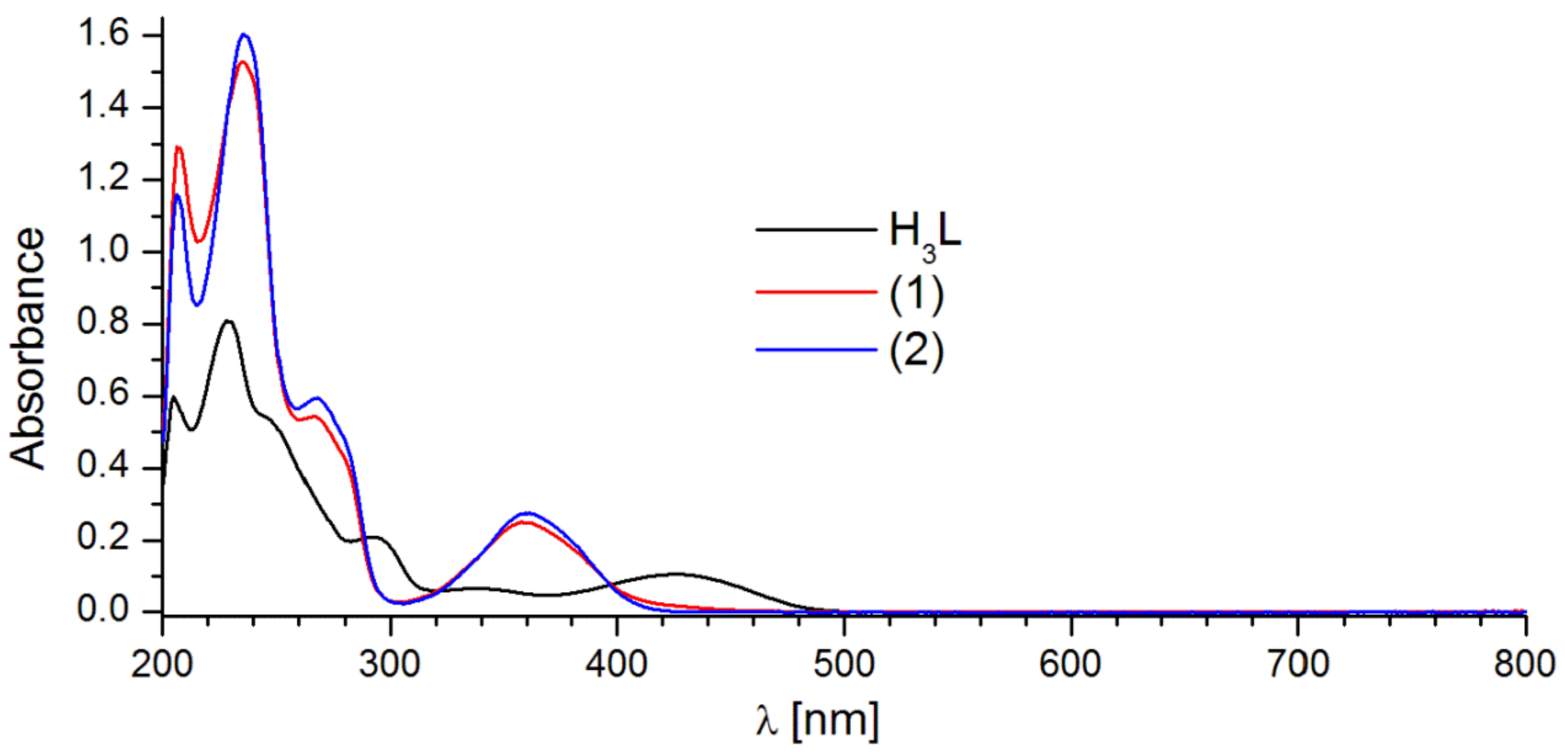
The absorbance spectra of **H_3_L** ligand and complexes **1** (Zn^II^–Nd^III^–Zn^II^) and **2** (Zn^II^–Sm^III^–Zn^II^) in methanol solution (~2·10^−5^ M).

The UV-VIS absorption spectra of the Schiff-base ligand (**H_3_L**) and its corresponding Zn^II^–Ln^III^–Zn^II^ heteronuclear complexes **1**–**5** were obtained in a MeOH solution at 298 K (Table 2). The electronic spectra of ligand and complexes are shown in Figure 4. The ligand shows four absorption bands with maxima (Table 2). The band with the greatest intensity at 229.0 nm is attributed to π-π* transition in the benzene ring. The weak peaks at 292.5 and 341.5 nm correspond to the π-π* transition of the phenol and C = N chromophore groups, whereas weak peak at 425.5 nm is ascribed to the n-π* electron transition in the ligand [43,44,45].

The spectral profiles of the absorption spectra for the heterotrinuclear complexes are similar (Figure 4 and Figure S4). A weak absorption band of the ligand observed at λ = 292.5 nm, in the spectra of all complexes disappears. All complexes displayed three bands at ca. 234, 267 and 359 nm due to ligand based or ligand to metal charge transfer (LMCT) [46]. The absorption peaks maxima of complexes are red-shifted in the Zn^II^–Ln^III^–Zn^II^ complexes which could be evidence of coordination between the ligand **H_3_L** and metal atoms. The luminescence spectra of the free ligand and **1**–**5** complexes in a MeOH solution (2 × 10^−5^ M) were recorded in the UV-VIS region at room temperature. As can be seen from Figure 4 and Figure S4, each of the Zn^II^–Ln^III^–Zn^II^ complexes displays an essentially identical absorption spectrum, with a broad peak at ca. 359 nm. The emission of Ln^III^ ions in complexes is observed, when energy absorbed by the ligand is transferred from a triplet state of the ligand to a resonance state of the Ln^III^ by an intramolecular energy transfer process. 

**Figure 5:**
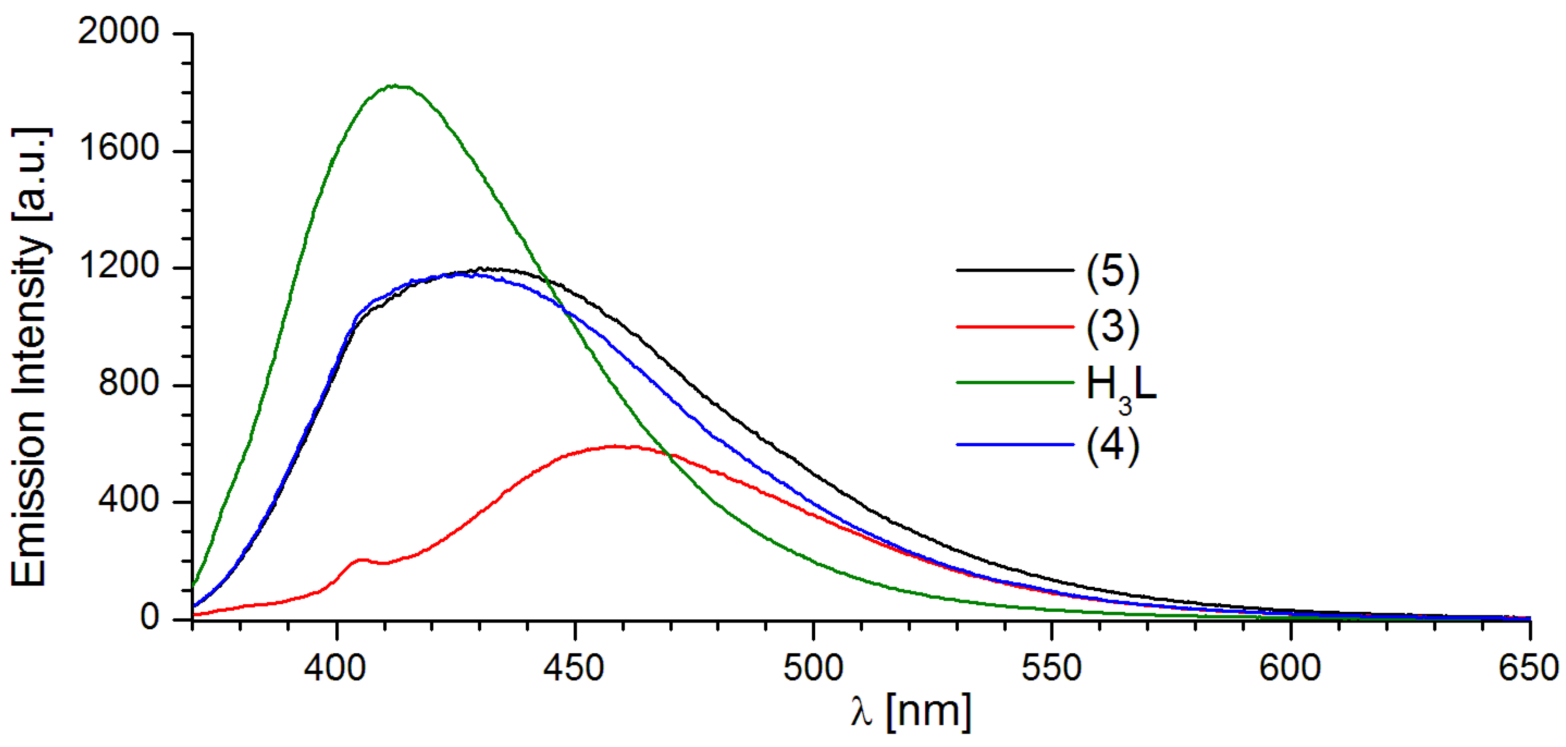
Luminescence spectra of **H_3_L** ligand and complexes **3** (Zn^II^–Eu^III^–Zn^II^), **4** (Zn^II^–Tb^III^–Zn^II^) and **5** (Zn^II^–Dy^III^–Zn^II^) in methanol solution (~2·10^−5^ M).

Excitation of the Schiff-base ligand at λ_ex_ = 337 nm produced luminescence with an emission maximum at λ_em_ = 414 nm which can be assigned to the π-π* electronic transition in the ligand. The corresponding emission spectra upon excitation at λ_ex_ = 359 nm are shown in Figure 5 and Figure S5. The emission spectra of **1** and **3**–**5** complexes present a broad emission band with an emission maximum at 421, 458, 424 and 430 nm respectively. The **H_3_L** ligand plays an important role in the emission spectra of these complexes (Figure 5 and Figure S5, Table 2). The emission spectra of all complexes are red shifted compared to the emission spectrum of the free ligand **H_3_L**. This is presumably attributed to the coordination effect of the ligand to the metal ions [42,46,47].

The emission spectra of complexes **3**, **4** and **5** don’t exhibit characteristic emission bands of f-f transition, implying that energy transfer from the ligand to Eu^III^, Tb^III^ and Dy^III^ ions was inefficient. Moreover, emission intensity of the complex **3** decreased obviously under the same conditions, as it was observed earlier [48]. It may be due to the thermal quenching of the ^5^D_0_ level of Eu^III^ by an intramolecular LMCT process [49]. 

**Figure 6:**
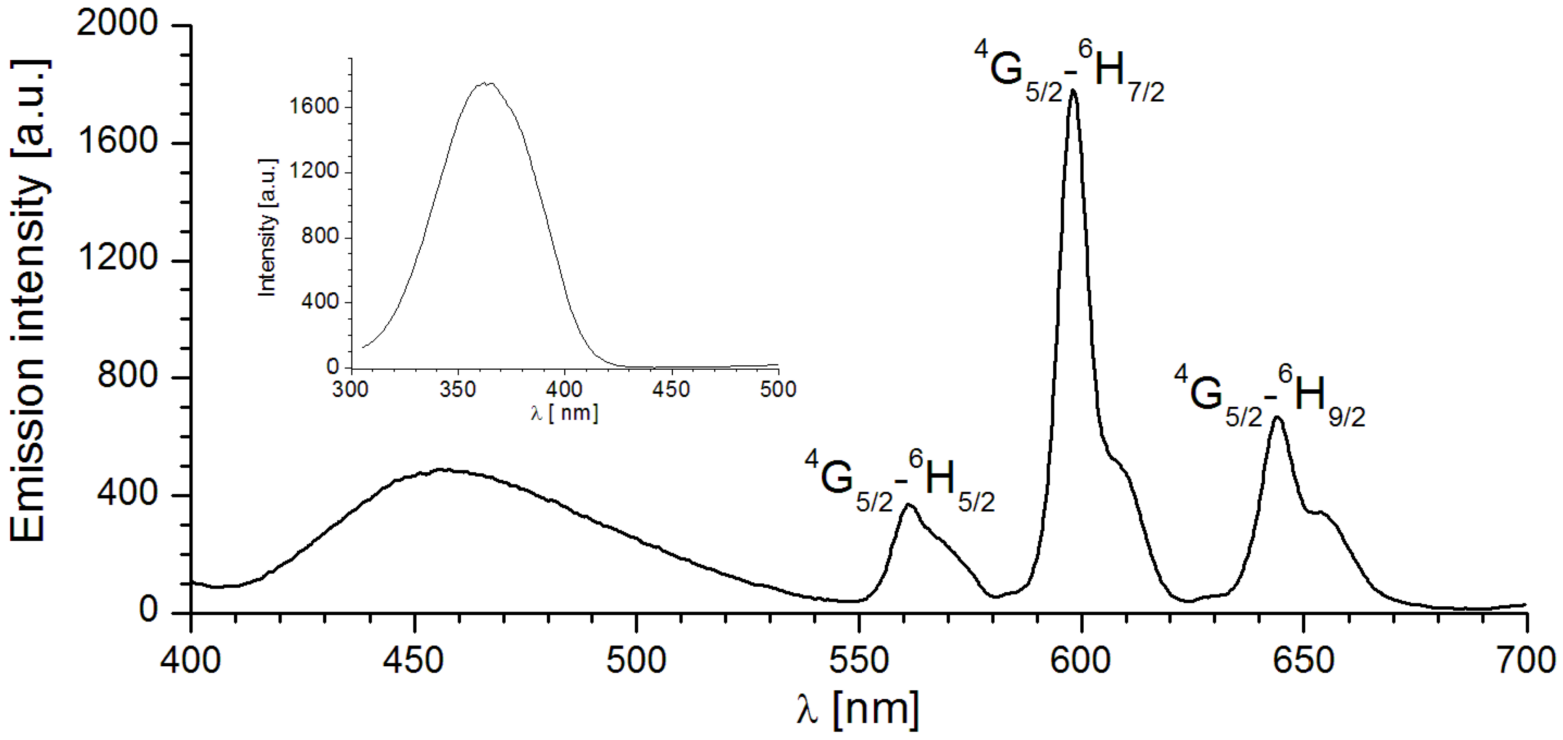
Photoluminescent spectrum of **2** (Zn^II^–Sm^III^–Zn^II^) in methanol solution; the inset is the excitation spectrum of the studied compound.

Excited at λ_ex_ = 359 nm (Figure 6, Table 2), complex **2** shows three characteristic emission bands of Sm^III^ ion at 561, 598 and 644 nm. The strongest emission was observed at 598 nm for a ^4^G_5/2_-^6^H_7/2_ transition [50,51]. Also, this spectrum shows a weak, broad band at 458 nm, which has been assigned to the π-π* transitions of the ligand **H_3_L**. It indicates that the intramolecular energy transfer process from the triplet state of the Schiff base ligand **H_3_L** to the emission levels of Ln^III^ ions was efficient in the Sm^III^ complex (**2**) only [52,53,54]. 

**Figure 7:**
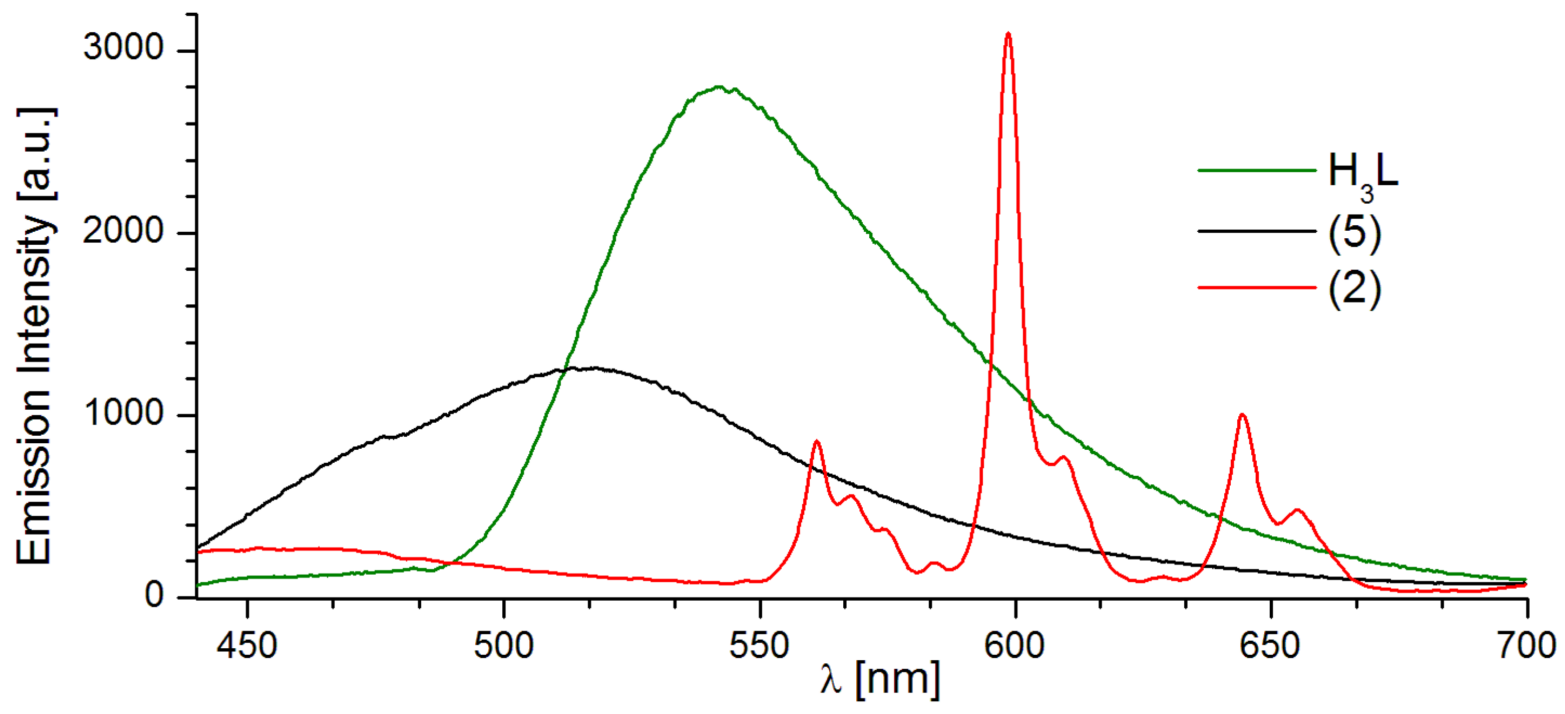
Solid state emission spectra of **H_3_L** ligand and complexes **2** (Zn^II^–Sm^III^–Zn^II^) and **5** (Zn^II^–Dy^III^–Zn^II^).

The luminescence properties of compounds **1**–**5** and the ligand **H_3_L** have been studied in the solid state. The intense broad emission bands at 541 nm for **H_3_L** ligand is observed with excitation wavelength of 363 nm (Figure 7 and Figure S6, Table 2). Upon excitation at 357 nm, compounds **3**–**5** show intense, broad emission bands, with the maximum peaks at 461, 507 and 519 nm for **3**–**5**, respectively, whereas for **1** and **2**, low emission at 460 and 457 nm was observed. Compared with the free **H_3_L** ligand, their emissions are blue shifted (similar as in Ref. [11]). These bands can be tentatively assigned to intra ligand luminescence. A much more shifts of emission in **3** (in solution and solid phase) may originate from the ligand-to-metal-charge transfer emission [48,49,52].

The luminescence behavior of the complex **2** with a linear arrangement of the Zn^II^–Sm^III^–Zn^II^ core in solid state is similar to that of **2** in methanol solution. This may arise from the less evident changes in the conformations between a solution and a solid state of **2** than in the compounds **3**–**5** crystallizing in trigonal space group with strained conformation of pseudo hexagonal symmetry and a bent layout of the trinuclear metal core. Excitation of complex **2** results in the luminescence of Sm^III^ at 561, 598 and 644 nm originating from the ^4^G_5/2_-level (Figure 7).

### 2.3. Magnetic Properties

**Figure 8:**
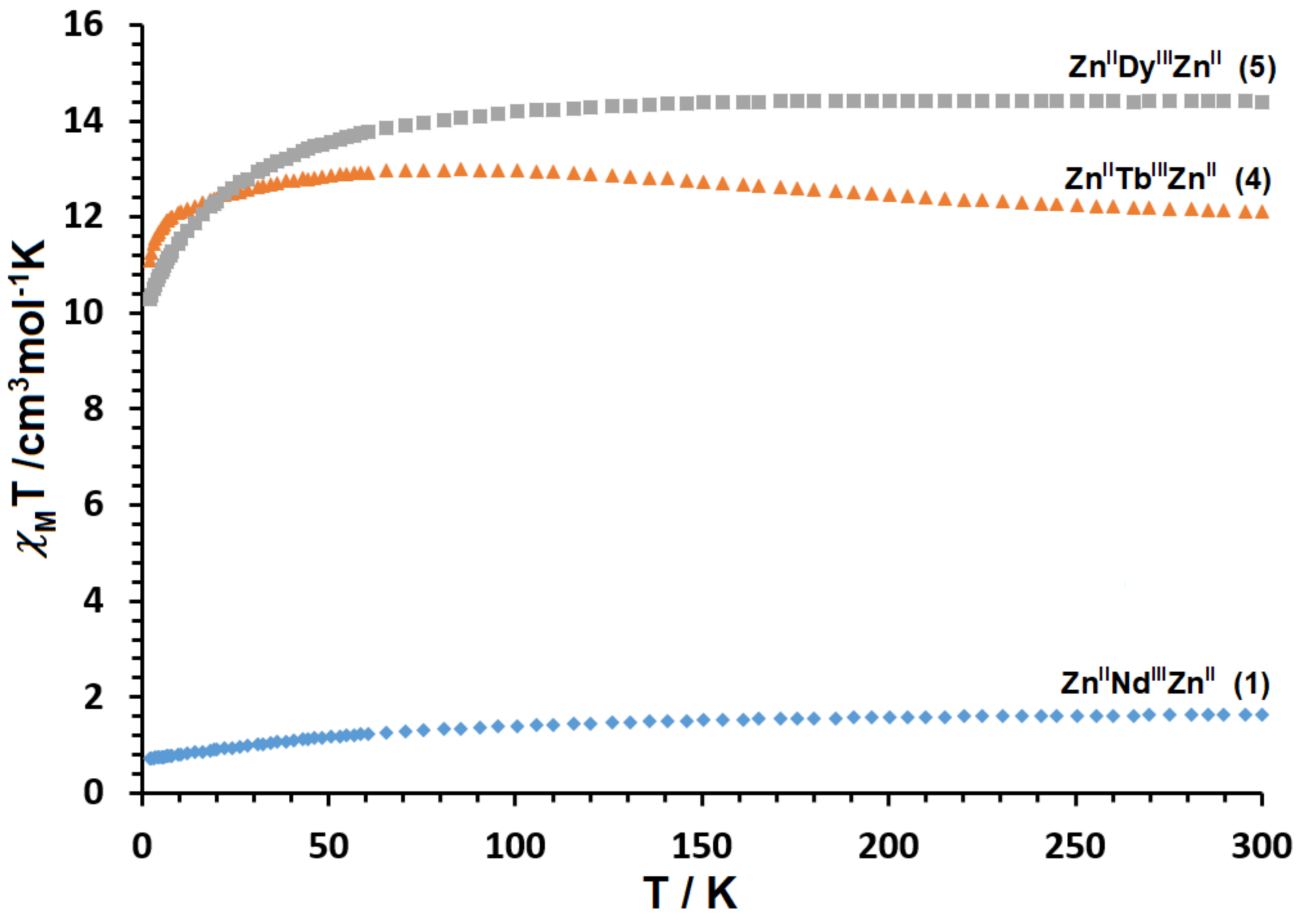
Temperature dependence of experimental *χ_M_T* versus *T* for **1** (Zn^II^–Nd^III^–Zn^II^), **4** (Zn^II^–Tb^III^–Zn^II^) and **5** (Zn^II^–Dy^III^–Zn^II^).

Temperature-dependent of molar susceptibility measurements of samples were carried out in an applied magnetic field of 0.1 T over the temperature range 1.8–300 K. The data are presented as plots of *χ_M_T* and *χ_M_*^−1^ versus *T* in Figure 8, Figures S7 and S8, where *χ_M_* is the molar magnetic susceptibility and *T* is the absolute temperature. The experimentally determined the *χ_M_T* values of compounds **1**, **4** and **5** at room temperature and *χ_M_T* values theoretically calculated by the following equation [55]:

(1) $$\chi_{M}T=((N\beta^{2}/3k)[g_{Ln}^{2}J_{Ln}\left(J_{Ln}+1)]\right)$$

where *N* is Avogadro constant, *β* is the Bohr magneton, *k* is Boltzman’s constant, *g_Ln_* is the *g* factor of the ground *J* terms of Ln^III^ and is expressed as: *g_Ln_* = 3/2 + [*S*(*S* + 1) − *L*(*L* + 1)]/2*J*(*J* + 1) are summarized in the Table S3.

At 300 K the determined *χ_M_T* values of 1.62 cm^3^Kmol^−1^ (**1**), 12.10 cm^3^Kmol^−1^ (**4**), 14.39 cm^3^Kmol^−1^ (**5**), respectively are compatible with those expected for Nd^III^ (^4^I_9/2_, *S* = 3/2, *L* = 6, *J* = 9/2), Tb^III^ (^7^F_6_, *S* = 3, *L* = 3, *J* = 6), Dy^III^ (^6^H_15/2_, *S* = 5/2, *L* = 5, *J* = 15/2) magnetically isolated metal ions. As shown in Figure 8 there is a smooth decrease of the *χ_M_T* product in the studied compounds from about 50 K to about 2 K for **2**, from 60 K to about 20 K for **4** and from 60 K to about 10 K for **5**, respectively. Finally, these values drop at 2.0 K down to 11.08 cm^3^Kmol^−1^ (**4**), and 10.28 cm^3^Kmol^−1^ (**5**), respectively. The reduction of *χ_M_T* values at the low temperature could mainly arise from the crystal field splitting of Ln^III^ ions and/or a weak interaction between magnetic centers of neighboring molecules [55,56,57]. In lanthanides the 4f orbitals are efficiently shielded and the influence of the neighboring groups on the magnetic properties is less evident than in 3d paramagnetic compounds. The magnetic properties of these ions are governed by strong orbital angular momentum contribution and the spin–orbit coupling that splits the ^2+1^L multiplets of the 4*f*^n^ ions into ^2*S*+1^L*_J_* J-states. Usually, for most of the trivalent rare-earths ions, the ^2+1^L*_J_* free ion ground state is well separated from the excited ones, thus at room and at lower temperatures the ground state is the only thermally populated one. As lanthanide ions are placed in a crystal field, the ^2+1^L*_J_* states are split into Stark components (up to 2*J* + 1, *n* = even; *J* + 1/2, *n* = odd). The magnetic behavior will then be governed by the thermal population of these sets of levels, preventing the application of a spin-only Hamiltonian for quantitative investigations of exchange interactions between lanthanide ions (only the Gd^III^ ion exhibits a quenched orbital momentum which can be therefore treated as a pure spin ground state) [55,56].

## 3. Materials and Methods

### 3.1. Materials

The chemicals: 5-bromo-2-hydroxy-3-methoxybenzaldehyde, 1,3-diamino-2-hydroxypropane, Zn(CH_3_COO)_2_·2H_2_O, Nd(NO_3_)_3_·6H_2_O, Sm(NO_3_)_3_·6H_2_O, Eu(NO_3_)_3_·5H_2_O, Tb(NO_3_)_3_·5H_2_O or Dy(NO_3_)_3_·xH_2_O and CH_3_OH (solvent) were of analytical reagent grade. They were purchased from commercial sources and used as received without further purification.

### 3.2. Synthesis of the ***H_3_L***

*N*,*N*′-bis(5-bromo-3-methoxysalicylidene)-1,3-diamino-2-propanol **H_3_L**, was synthesized by the 2:1 condensation reaction between 5-bromo-2-hydroxy-3-methoxybenzaldehyde (2.31 g, 10 mmol) and 1,3-diamino-2-hydroxypropane (0.45 g, 5 mmol) in hot methanol (100 mL) following the procedure reported in the literature [58]. The compound was separated as yellow needles and recrystallized twice from methanol. The empirical formula and the molecular weight are C_19_H_20_Br_2_N_2_O_5_ and 516.18 g/mol, respectively. Yield 73%. Analytical data (%), Calcd: C, 44.21; H, 3.91; N, 5.43. Found: C, 44.10; H, 3.80; N, 5.10. IR (ATR); 3285 m, 2961 w, 1637 vs, 1518 s, 1478 s, 1460 s, 1438 m, 1373 w, 1338 w, 1248 vs, 1229 s, 1210 s, 1104 s, 1018 w, 970 m, 949 m, 924 m, 865 s, 845 s, 830 s, 760 s, 697 m, 637 s, 573 w. ^1^H NMR (500 MHz, CDCl_3_, δ): 3.76 (dd, *J* = 12.3, 6.6 Hz, 2H, CH_2_), 3.89 (dd, *J* = 12.6, 4.4 Hz, 2H, CH_2_), 3.90 (s, 6H, OCH_3_), 4.28 (m, 1H, CH), 7.00 (d, *J* = 2.2 Hz, 2H, CH), 7.03, (d, *J* = 2.2 Hz, 2H, CH), 8.32 (s, 2H, CH), 13.65 (bs, 2H, OH). ^13^C NMR (126 MHz, DMSO-d_6_, δ): 56.06, 60.86, 68.93, 107.05, 116.82, 118.53, 124.89, 150.00, 154.22, 166.10.

### 3.3. Synthesis of the Zn^II^–Ln^III^–Zn^II^ Complexes

The heterotrinuclear compounds **1**–**5** were prepared as follows: Zn(CH_3_COO)_2_·2H_2_O (0.4 mmol, 0.0878 g) dissolved in methanol (10 mL) was added dropwise to the stirred solution of the Schiff base **H_3_L** (0.4 mmol, 0.2065 g) in methanol (100 mL) to produce a yellow coloured solution. The reaction mixture was stirred for 30 min at 45 °C. Next, the freshly prepared solution of Nd(NO_3_)_3_·6H_2_O (0.2 mmol, 0.0877 g), Sm(NO_3_)_3_·6H_2_O (0.2 mmol, 0.0889 g), Eu(NO_3_)_3_·5H_2_O (0.2 mmol, 0.0856 g), Tb(NO_3_)_3_·5H_2_O (0.2 mmol, 0.0870 g) or Dy(NO_3_)_3_·xH_2_O (0.2 mmol, 0.0875 g) in methanol (5 mL) was added slowly to the mixture with constant stirring. The resulting deep yellow solution was stirred for another 30 min and left undisturbed. Yellow crystals of a shape of prims (**1**, **2**) or hexagonal plates (**3**–**5**) suitable for single crystal X-ray diffraction formed at 4 °C after two weeks.

#### 3.3.1. [Zn_2_Nd(ac)_2_(HL)_2_]NO_3_·3H_2_O (**1**)

C_42_H_48_N_5_O_20_Br_4_Zn_2_Nd, *M* = 1537.47 g/mol. Yield 32%. Analytical data (%), Calcd: C, 32.81; H, 3.15; N, 4.56; Zn, 8.51; Nd, 9.38. Found: C, 32.50; H, 3.00; N, 4.20; Zn, 8.30; Nd, 9.10. IR (ATR); 3315 w, 1628 s, 1549 s, 1465 s, 1436 vs, 1402 s, 1344 w, 1293 vs, 1232 vs, 1213 vs, 1136 w, 1097 m, 1055 m, 951 m, 844 s, 786 s, 758 m, 690 s, 665 s, 614 m, 571 m, 556 w, 540 w. **Crystal Data**: monoclinic, space group *P*2_1_/*n* (no. 14), *a* = 9.9805(4) Å, *b* = 24.1307(11) Å, *c* = 22.9091(7) Å, *β* = 91.549(3)°, *V* = 5515.4(4) Å^3^, *Z* = 4, *T* = 293.0 K, μ(MoKα) = 4.763 mm^−1^, *Dcalc* = 1.852 g/cm^3^, 41616 reflections measured (5.3° ≤ 2Θ ≤ 50.48°), 9953 unique (*R*_int_ = 0.0806, R_sigma_ = 0.0852) which were used in all calculations. The final *R*_1_ was 0.0593 (I >= 2σ(I)) and *wR*_2_ was 0.1544 (all data). CCDC No: 1849667.

#### 3.3.2. [Zn_2_Sm(ac)_2_(HL)_2_]NO_3_·3CH_3_OH·0.3H_2_O (**2**)

C_45_H_54.67_N_5_O_20.33_Br_4_Zn_2_Sm, *M* = 1591.07 g/mol. Yield 30%. Analytical data (%), Calcd: C, 33.96; H, 3.46; N, 4.40; Zn, 8.22; Sm, 9.45. Found: C, 34.10; H, 3.50; N, 4.10; Zn, 8.00; Sm, 9.10. 

IR (ATR); 3180 w, 1629 vs, 1570 m, 1554 s, 1464 m, 1438 s, 1402 m, 1290 s, 1234 m, 1214 s, 1100 m, 1055 m, 1035 w, 952 m, 846 s, 785 m, 755 s, 692 m, 669 m, 615 m, 570 w, 540 w. **Crystal Data**: monoclinic, space group *P*2_1_/*n* (no. 14), *a* = 10.26745(18) Å, *b* = 23.8581(3) Å, *c* = 22.5353(3) Å, *β* = 91.2611(13)°, *V* = 5518.94(14) Å^3^, *Z* = 4, *T* = 120.01(10) K, μ(CuKα) = 12.967 mm^−1^, *Dcalc* = 1.915 g/cm^3^, 38,779 reflections measured (7.42° ≤ 2Θ ≤ 152.88°), 11,370 unique (*R*_int_ = 0.0548, R_sigma_ = 0.0444) which were used in all calculations. The final *R*_1_ was 0.0481 (I >= 2σ(I)) and *wR*_2_ was 0.1384 (all data). CCDC No: 1849666.

#### 3.3.3. [Zn_2_Eu(ac)_2_(HL)_2_]NO_3_·5.33H_2_O (**3**)

C_42_H_52.67_N_5_O_22.33_Br_4_Zn_2_Eu, *M* =1587.23 g/mol). Yield 26%. Analytical data (%), Calcd: C, 31.78; H, 3.34; N, 4.41; Zn, 8.24; Eu, 9.57. Found: C, 31.90; H, 3.10; N, 4.20; Zn, 8.30; Eu, 9.30. IR (ATR); 3192 w, 1632 s, 1570 m, 1462 s, 1435 m, 1402 m, 1344 w, 1293 vs, 1232 vs, 1213 vs, 1136 w, 1097 m, 1055 m, 951 m, 844 s, 786 s, 758 m, 690 s, 665 s, 614 m, 571 m, 556 w, 540 w. **Crystal Data**: trigonal, space group *R*-3*c* (no. 167), *a* = 19.8962(4) Å, *c* = 76.6553(19) Å, *V* = 26279.2(9) Å^3^, *Z* = 18, *T* = 119.99(10) K, μ(CuKα) = 12.414 mm^−1^, *Dcalc* = 1.805 g/cm^3^, 58409 reflections measured (8.88° ≤ 2Θ ≤ 135.34°), 5291 unique (*R*_int_ = 0.1573, R_sigma_ = 0.0539) which were used in all calculations. The final *R*_1_ was 0.0785 (I >= 2σ(I)) and *wR*_2_ was 0.2254 (all data). CCDC No: 1849668.

#### 3.3.4. [Zn_2_Tb(ac)_2_(HL)_2_]NO_3_·5.33H_2_O (**4**)

C_42_H_52.67_N_5_O_22.33_Br_4_Zn_2_Tb, *M* = 1594.19 g/mol. Yield 26%. Analytical data (%), Calcd: C, 31.64; H, 3.33; N, 4.39; Zn, 8.2; Tb, 9.97. Found: C, 31.40; H, 3.50; N, 4.20; Zn, 8.10; Tb, 9.70. IR (ATR); 3352 w, 1629 s, 1579 m, 1554 m, 1439 w, 1402 m, 1348 w, 1308 w, 1289 vs, 1236 s, 1216 s, 1139 w, 1099 w, 1056 m, 949 m, 846 m, 787 m, 755 s, 693 s, 670 s, 615 s, 577 w, 553 w, 540 w. **Crystal Data**: trigonal, space group *R*-3*c* (no. 167), *a* = 20.0572(5) Å, *c* = 76.388(3) Å, *V* = 26613.4(12) Å^3^, *Z* = 18, *T* = 293.0 K, μ(MoKα) = 4.765 mm^−1^, *Dcalc* = 1.790 g/cm^3^, 70659 reflections measured (4.8° ≤ 2Θ ≤ 50.48°), 5349 unique (*R*_int_ = 0.0860, R_sigma_ = 0.0376) which were used in all calculations. The final *R*_1_ was 0.0561 (I >= 2σ(I)) and *wR*_2_ was 0.1704 (all data). CCDC No: 1849669.

#### 3.3.5. [Zn_2_Dy(ac)_2_(HL)_2_]NO_3_·5.33H_2_O (**5**)

C_42_H_52.67_N_5_O_22.33_Br_4_Zn_2_Dy, *M* = 1597.77 g/mol. Yield 26%. Analytical data (%), Calcd: C, 31.57; H, 3.32; N, 4.38; Zn, 8.19; Dy, 10.17. Found: C, 31.70; H, 3.40; N, 4.20; Zn, 8.30; Dy, 10.40. IR (ATR); 3182 w, 1629 s, 1570 m, 1554 m, 1466 m, 1439 m, 1402 m, 1289 vs, 1234 s, 1214 s, 1100 m, 1055 m, 1035 m, 950 m, 846 s, 784 s, 754 s, 692 m, 669 s, 615 s, 571 w, 540 w. **Crystal Data**: trigonal, space group *R*-3*c* (no. 167), *a* = 19.8706(5) Å, *c* = 76.007(2) Å, *V* = 25990.1(12) Å^3^, *Z* = 18, *T* = 119.95(10) K, μ(CuKα) = 11.701 mm^−1^, *Dcalc* = 1.838 g/cm^3^, 35,163 reflections measured (8.9° ≤ 2Θ ≤ 135.36°), 5229 unique (*R*_int_ = 0.0842, R_sigma_ = 0.0405) which were used in all calculations. The final *R*_1_ was 0.0610 (I >= 2σ(I)) and *wR*_2_ was 0.1699 (all data). CCDC No: 1849665.

### 3.4. Methods

The contents of carbon, hydrogen and nitrogen in the complexes were determined by elemental analysis using a CHN 2400 Perkin Elmer analyser. The contents of zinc and lanthanides were established using ED XRF spectrophotometer (Canberra-Packard, Schwadorf, Austria). The infrared spectra were acquired using a Thermo Scientific Nicolet 6700 FTIR with a Smart iTR diamond ATR accessory. Data was collected between 4000 cm^−1^ and 520 cm^−1^, with a resolution of 4 cm^−1^ for 16 scans. The NMR spectra were recorded on Bruker Avance 500 MHz in CDCl_3_ and DMSO-d_6_ solutions. Magnetic susceptibility measurements were performed on finely ground crystalline samples over the temperature range 1.8–300 K at magnetic field 0.1 T using a Quantum Design SQUID-VSM magnetometer. Corrections are based on subtracting the sample—holder signal and contribution χ_D_ estimated from the Pascal’s constants [55,59]. 

The electronic absorption spectra of the ligand **H_3_L** and complexes **1**–**5** were recorded with a Shimadzu UV-2401 PC 2001 spectrophotometer, in 10 × 10 mm quartz cells, in methanol at concentration about 2·10^−5^ mol/L. Excitation and emission spectra of the studied systems, in methanol solution and in solid state, were measured at ambient temperature and UV-VIS range used a Hitachi F-7000 fluorescence spectrophotometer. For accuracy of data, the solid state samples were measured within the same sample holder to ensure the consistent amount of luminescent materials in all samples. Excitation and emission spectra were corrected for the instrumental response. All measurements were carried out under the same experimental conditions. 

Diffraction data were collected at room temperature (**1**, **4**) on Oxford Diffraction Xcalibur CCD diffractometer with graphite-monochromated MoK α radiation (λ = 0.71073 Å) using the ω scan technique and at 120(1) K (**2**, **3**, **5**) on SuperNova X-ray diffractometer equipped with Atlas S2 CCD detector using the mirror-monochromatized CuK*α* radiation (λ = 1.54184 Å). The diffraction data resolution was 0.79 Å for **2** and 0.83 Å for **1**,**3**–**5**. Absorption effects were corrected with CrysAlisPro 1.171.38.46 [60] by empirical absorption correction using spherical harmonics, implemented in SCALE3 ABSPACK scaling algorithm for **1** and **4**, and by analytical numeric absorption correction using a multifaceted crystal model based on expressions derived by R.C. Clark & J.S. Reid for **2**, **3**, **5** [61]. The structures were solved by direct methods using the ShelXT [62] structure solution program using intrinsic phasing and refined with the Olex2.refine refinement package using Gauss-Newton minimisation [63]. The lanthanide ions in **3**–**5** lie on a 2-fold axis with sof = 0.5. In **3**–**5** the nitrate ion occupies a special position at a 3-fold rotoinversion axis with sof 1/3 and a position near the coordination unit which is shared with a solvent molecule with sof’s 1/6. Therefore, for nitrate ions in **3**–**5** the bond length restraints were applied by DFIX instructions to 1.22(1) and DANG 2.10(2) Å for *N*–*O* and *O*–*O* distances, respectively. Non-hydrogen atoms with except of nitrate N and O atoms and solvent molecules were refined with anisotropic displacement parameters. The C-bound H atoms were positioned geometrically and the ‘riding’ model for the C–H bonds was used in the refinement. The summary of experimental details and the crystal structure refinement parameters are given in Table 1 and Table S2. The experimental details and final atomic parameters have been deposited with the Cambridge Crystallographic Data Centre as supplementary material (CCDC: 1849665–1849669). Copies of the data can be obtained free of charge on request via www.ccdc.cam.ac.uk/data_request/cif, or by emailing data_request@ccdc.cam.ac.uk.

## 4. Conclusions

A new family of trinuclear Zn^II^–Ln^III^–Zn^II^ complexes have been synthesized and characterized by single-crystal X-ray diffraction, IR and elemental analyses. The luminescent and magnetic properties of the complexes were studied. The two *μ*-acetato anions arranged in a *cisoid* manner are responsible for the chirality of the coordination units. All the studied crystals **1**–**5** are racemic compounds. The chirality of the coordination units influences the structure of the outer coordination spheres. The hydroxyl group at the propyl linkage does not participate in coordination in **1**–**5** but it forms hydrogen bonds with anions or solvent molecules blocking the direct access to the metal ions. In this way there are formed two hydrophilic pockets accessible for hydrogen bonded small molecules. There are no inner-sphere-coordinated solvent molecules or nitrate anions, which is beneficial for luminescent properties. Different conformation of coordination units in crystals **1**, **2** and **3**–**5** influences the linearity of the metal arrangement Zn^II^–Ln^III^–Zn^II^ and the mutual orientation of Zn–O_apical_ vectors, which may be important for the physicochemical properties.

The complexes **1**, **3**–**5** containing Nd^III^, Eu^III^, Tb^III^ and Dy^III^ ions show a blue emission attributed to the emission of the ligand **H_3_L**. The characteristic emission bands of f-f* transitions were observed for the Sm^III^ complex **2** with a linear arrangement of Zn^II^–Sm^III^–Zn^II^ metal ions. 

The magnetic properties for Zn^II^–Ln^III^–Zn^II^ compounds **1**, **4**, **5** are characteristic for the paramagnetism of the corresponding lanthanide(III) ions. 

The results will be employed in the further investigation as a reference for the future studies concerning structural *versus* luminescent and magnetic properties of homo- and heteronuclear coordination compounds.

## Figures and Tables

**Table 1:** Selected structural parameters ^a^ for complexes **1**–**5**.

Parameter.	1	2	Parameter	3	4	5
Ln1–Zn1	3.513(1)	3.5268(7)	Ln1–Zn1	3.458(1)	3.4242(9)	3.414(1)
Ln1–Zn2	3.556(1)	3.4915(7)	Ln1–O1	2.354(5)	2.330(5)	2.367(4)
Ln1–O1	2.472(5)	2.414(3)	Ln1–O2	2.409(5)	2.386(5)	2.303(4)
Ln1–O2	2.442(5)	2.414(3)	Ln1–O3	2.637(6)	2.649(5)	2.879(4)
Ln1–O3	2.825(6)	2.650(4)	Ln1–O4	2.856(5)	2.905(5)	2.612(5)
Ln1–O4	2.729(6)	2.704(3)	Ln1–O7	2.375(5)	2.345(5)	2.324(4)
Ln1–O6	2.442(5)	2.412(3)	Zn1–O1	2.056(6)	2.064(5)	2.094(4)
Ln1–O7	2.457(5)	2.440(3)	Zn1–O2	2.082(5)	2.089(5)	2.054(5)
Ln1–O8	2.671(5)	2.689(4)	Zn1–O6	1.997(6)	2.001(5)	1.988(5)
Ln1–O9	2.730(6)	2.803(4)	Zn1–N1	2.082(7)	2.083(7)	2.034(6)
Ln1–O12	2.437(6)	2.369(4)	Zn1–N2	2.035(7)	2.049(7)	2.092(6)
Ln1–O13	2.384(6)	2.407(4)	Zn1–O1–Ln1	103.0(1)	102.2(2)	103.0(2)
Zn1–O1	2.067(5)	2.063(3)	Zn1–O2–Ln1	100.4(1)	99.6(2)	99.7(2)
Zn1–O2	2.044(5)	2.077(3)	Δ ^b^ (Zn1)	0.48	0.49	0.48
Zn1–O11	1.996(6)	2.005(4)	*σ*^c^ (Zn1)	35.5	36.0	34.9
Zn1–N1	2.022(8)	2.075(4)	*ω* ^d^	62.4(4)	61.6(3)	62.2(2)
Zn1–N2	2.084(7)	2.069(4)	*ε* ^e^	62.0(3)	61.3(4)	62.0(3)
Zn2–O6	2.064(5)	2.059(4)	*φ* ^f^	173.3(2)	172.8(1)	172.9(1)
Zn2–O7	2.068(5)	2.077(3)				
Zn2–O14	2.009(6)	1.993(4)				
Zn2–N3	2.052(8)	2.079(5)				
Zn2–N4	2.074(6)	2.050(4)				
Zn1–O1–Ln1	101.1(2)	103.7(1)				
Zn1–O2–Ln1	102.7(2)	103.3(1)				
Zn2–O6–Ln1	103.9(2)	102.4(1)				
Zn2–O7–Ln1	103.3(2)	100.9(1)				
Δ ^b^ (Zn1; Zn2)	0.46; 0.50	0.51; 0.49				
*σ*^c^ (Zn1; Zn2)	34.7; 34.8	34.5; 35.7				
*ω* ^d^	36.2(4)	34.6 (3)				
*ε* ^e^	42.6(4)	42.1(3)				
*φ* ^f^	178.8(1)	178.8(3)				

^a^ Distances in Å, angles in deg, Ln = Nd (**1**), Sm (**2**), Eu (**3**), Tb (**4**), Dy (**5**), respectively; ^b^ Δ–deviation of Zn^II^ ion from the N_2_O_2_ mean plane; ^c^ *σ*—dihedral angle between ZnO_(phenoxo)2_ and LnO_(phenoxo)2_ planes; ^d^ *ω*—torsion angle between two Zn–O vectors within the coordination unit O11–Zn1–Zn2–O14 in **1** and **2** and the analogous torsion O6–Zn1–Zn1*^i^*–O6*^i^* (symmetry code: *^I^* 1/3+x-y, 2/3-y, 1/6-z) in **3–5**; ^e^ *ε*—torsion angle between two N_2_O_2_ mean planes in the coordination core; ^f^ *φ*—valence angle along the Zn^II^–Ln^III^–Zn^II^ line.

**Table 2:** Photophysical data of **H_3_L** and complexes **1**–**5**.

Compound	λ_abs_^a^ (nm)	λ_ex_/λ_em_^a^ (nm)	λ_ex_/λ_em_^b^ (nm)
**H_3_L**	229.0; 292.5; 341.5; 425.5	337/414.0	363/541.0
**1**	235.5; 267.0; 358.5	359/421.0	357/457.0
**2**	235.5; 268.0; 359.5	359/458.0; 561.0; 598.0; 644.0	357/460.0; 561.0; 598.0; 644.0
**3**	234.5; 267.5. 359.0	359/458.0	357/461.0
**4**	236.0. 267.0; 359.0	359/424.0	357/507.0
**5**	234.0; 267.5; 359.5	359/430.0	357/519.0

^a^ in methanol solution, ^b^ in solid state.

## Supplementary Materials

The following are available online, **Table S1.** Selected coordination compounds with Schiff base ligand having 2-hydroxypropyl bridge. Involvement of the 2-hydroxypropyl bridge in the formation of coordination bond, **Table S2.** Crystallographic data for complexes **1**–**5**, **Figures S1**–**S3**. Location of hydrogen bonded NO_3_^−^ anion and water molecule in pockets formed by 2-hydroxypropyl groups in complexes Zn^II^–Nd^III^–Zn^II^ (**1**), Zn^II^–Sm^III^–Zn^II^ (**2**) and Zn^II^–Eu^III^–Zn^II^ (**3**), respectively, **Figure S4.** The absorbance spectra of compounds Zn^II^–Eu^III^–Zn^II^ (**3**), Zn^II^–Tb^III^–Zn^II^ (**4**) and Zn^II^–Dy^III^–Zn^II^ (**5**) in methanol solution (~2·10^−5^ M), **Figure S5.** Luminescence spectrum of complex **1** (Zn^II^–Nd^III^–Zn^II^) in methanol solution (~2·10^−5^ M), **Figure S6.** Solid state emission spectra of complexes **3** (Zn^II^–Eu^III^–Zn^II^) and **4** (Zn^II^–Tb^III^–Zn^II^), **Figure S7.** Temperature dependence of the experimental *χ*_M_^−1^ versus *T* for compound Zn^II^–Nd^III^–Zn^II^ (**1**), **Figure S8.** Temperature dependence of the experimental *χ*_M_^−1^ versus *T* for compounds Zn^II^–Tb^III^–Zn^II^ (**4**) and Zn^II^–Dy^III^–Zn^II^ (**5**), **Table S3.** Theoretical and experimental values of the *χ*_M_*T* product for compounds **1**, **4**, **5** at the room temperature, **Figure S9.** ^1^H NMR spectrum of ligand **H_3_L** in CDCl_3_ solution. **Figure S10.** ^13^C NMR spectrum of ligand **H_3_L** in DMSO-d_6_ solution.
