# Supplementary material for: Heterometallic ZnII–LnIII–ZnII Schiff Base Complexes with Linear or Bent Conformation—Synthesis, Crystal Structures, Luminescent and Magnetic Characterization
Source: Molecules. 2018 Jul 18;23(7):1761. doi: 10.3390/molecules23071761 (PMC6099393; doi:10.3390/molecules23071761)
Supplement: Supplementary file 1 [file molecules-23-01761-s001.zip › Supplementary Materials.pdf]

Supplementary Materials

Molecules

Heterometallic $Zn^{II}–Ln^{III}–Zn^{II}$ Schiff base complexes with linear or bent conformation – synthesis, crystal structures, luminescent and magnetic characterization

Barbara Miroslaw [1,*], Beata Cristóvão [2] and Zbigniew Hnatejko [3]

[1] Department of Crystallography, Faculty of Chemistry, Maria Curie-Sklodowska University, Maria Curie-Sklodowska sq. 3, 20-031 Lublin, Poland

[2] Department of General and Coordination Chemistry, Faculty of Chemistry, Maria Curie-Sklodowska University, Maria Curie-Sklodowska sq. 2, 20-031 Lublin, Poland; beata.cristovao@poczta.umcs.lublin.pl

[3] Department of Rare Earths, Faculty of Chemistry, Adam Mickiewicz University, Umultowska 89b, 61-614 Poznań, Poland; zbychuh@amu.edu.pl

* Correspondence: barbara.miroslaw@umcs.lublin.pl; Tel.: +48-81-537-55-82

List of items:

- **Table S1.** Selected coordination compounds with Schiff base ligand having 2-hydroxypropyl bridge. Involvement of the 2-hydroxypropyl bridge in the formation of coordination bond.
- **Table S2.** Crystallographic data for complexes **1-5**.
- **Figure S1.** Location of hydrogen bonded NO_3^- anion and water molecule in pockets formed by 2-hydroxypropyl groups in complex $Zn^{II}–Nd^{III}–Zn^{II}$ (**1**).
- **Figure S2.** Location of hydrogen bonded NO_3^- anion and methanol molecule in pockets formed by 2-hydroxypropyl groups in complex $Zn^{II}–Sm^{III}–Zn^{II}$ (**2**).
- **Figure S3.** Location of disordered hydrogen bonded NO_3^- anion and water molecule in pockets formed by 2-hydroxypropyl groups in complex $Zn^{II}–Eu^{III}–Zn^{II}$ (**3**).
- **Figure S4.** The absorbance spectra of compounds $Zn^{II}–Eu^{III}–Zn^{II}$ (**3**), $Zn^{II}–Tb^{III}–Zn^{II}$ (**4**) and $Zn^{II}–Dy^{III}–Zn^{II}$ (**5**) in methanol solution ($\sim 2\cdot 10^{-5}$ M).
- **Figure S5.** Luminescence spectrum of complex **1** ($Zn^{II}–Nd^{III}–Zn^{II}$) in methanol solution ($\sim 2\cdot 10^{-5}$ M).
- **Figure S6.** Solid state emission spectra of complexes **3** ($Zn^{II}–Eu^{III}–Zn^{II}$) and **4** ($Zn^{II}–Tb^{III}–Zn^{II}$).
- **Figure S7.** Temperature dependence of the experimental χ_M^{-1} versus T for $Zn^{II}–Nd^{III}–Zn^{II}$ (**1**).
- **Figure S8.** Temperature dependence of the experimental χ_M^{-1} versus T for $Zn^{II}–Tb^{III}–Zn^{II}$ (**4**) and $Zn^{II}–Dy^{III}–Zn^{II}$ (**5**).
- **Table S3.** Theoretical and experimental values of the $\chi_M T$ product for compounds **1**, **4**, **5** at the room temperature.
- **Figure S9.** ^1H NMR spectrum of ligand **H_3L** in $CDCl_3$ solution.
- **Figure S10.** ^{13}C NMR spectrum of ligand **H_3L** in DMSO-d_6 solution.

Table S1. Selected coordination compounds with Schiff base ligand having 2-hydroxypropyl bridge. [1]Involvement of the 2-hydroxypropyl bridge in the formation of coordination bond.

Structure	Refcode	Nuclearity	Metals	OH group[1]	Ref.
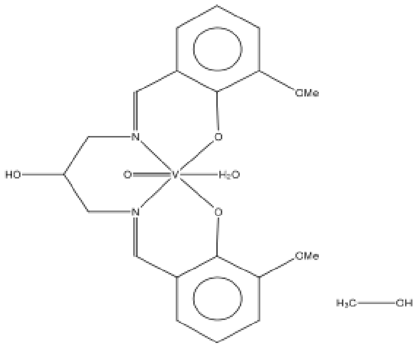	ABOZUP	1	V	No	[1]
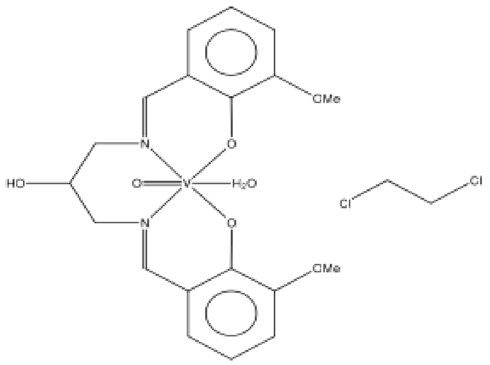	BEGHEB	1	V	No	[2]
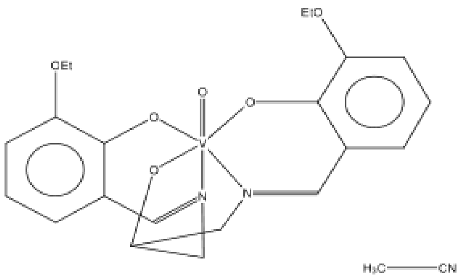	FEKHEJ	1	V	Yes	[3]
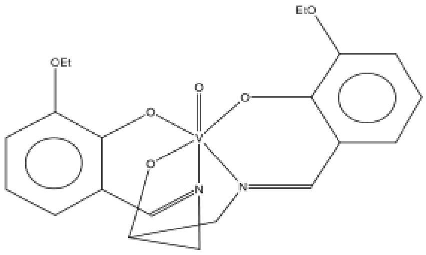	FEKHIN	1	V	Yes	[3]
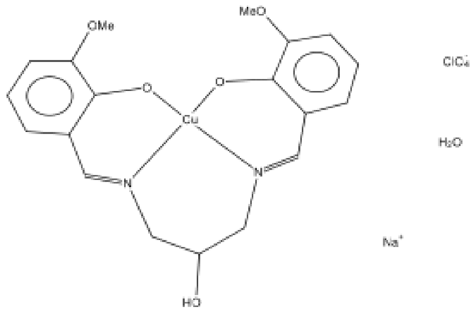	TIYYUX	2	CuNa	No	[4]

Structure	Refcode	Nucleārity	Metals	OH group[1]	Ref.
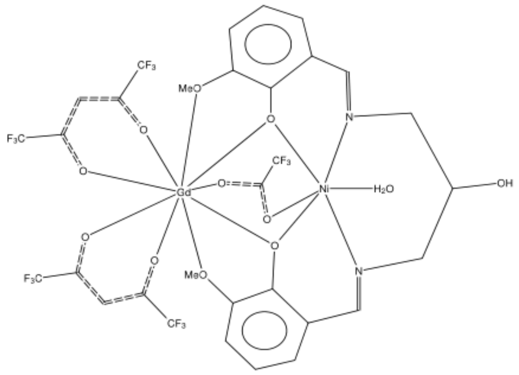	JAZFOI01	2	NiGd	No	[5]
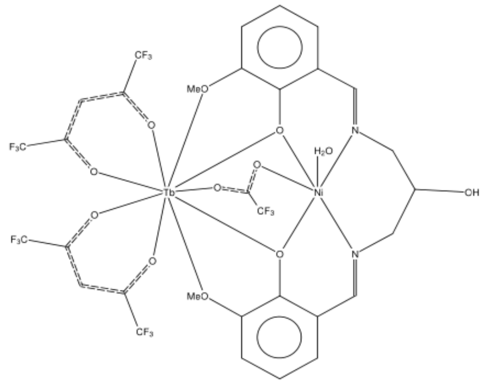	JAZFUO01	2	NiTb	No	[5]
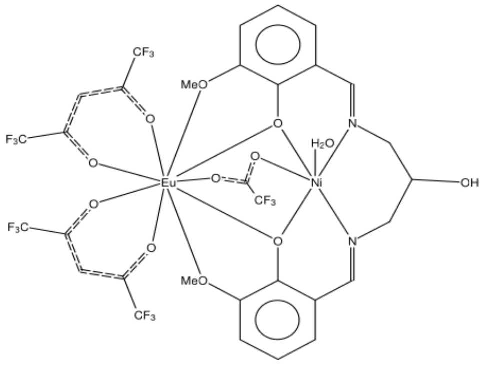	JAZGAV01	2	NiEu	No	[5]
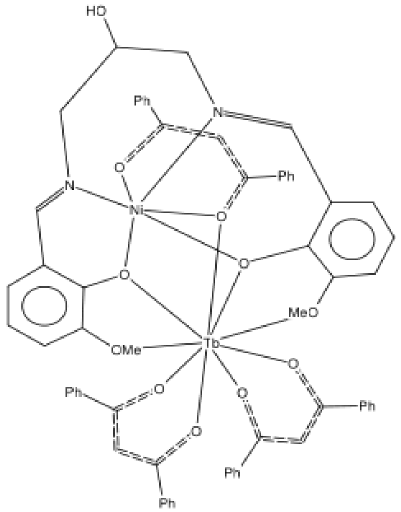	YECKOJ	2	NiTb	No	[5]

Structure	Refcode	Nuclearity	Metals	OH group[1]	Ref.
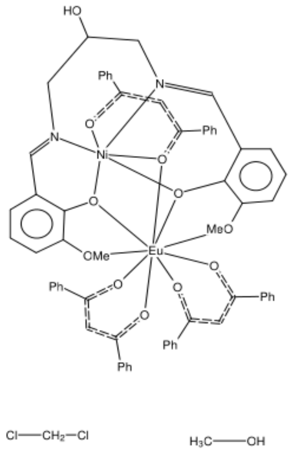	YECKUP	2	NiEu	No	[5]
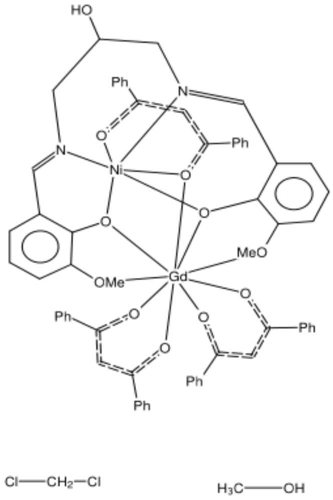	YECLAW	2	NiGd	No	[5]
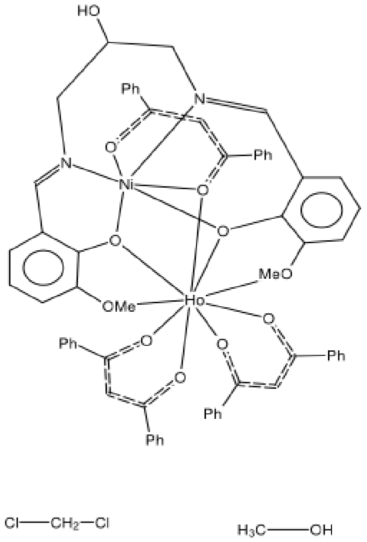	YECLEA	2	NiHo	No	[5]

Structure	Refcode	Nuclearity	Metals	OH group[1]	Ref.
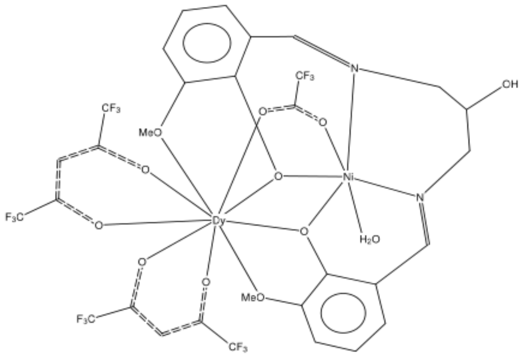	YECLIE	2	NiDy	No	[5]
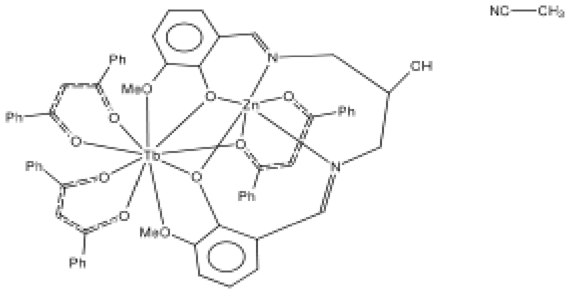	CANDUT	2	ZnTb	No	[6]
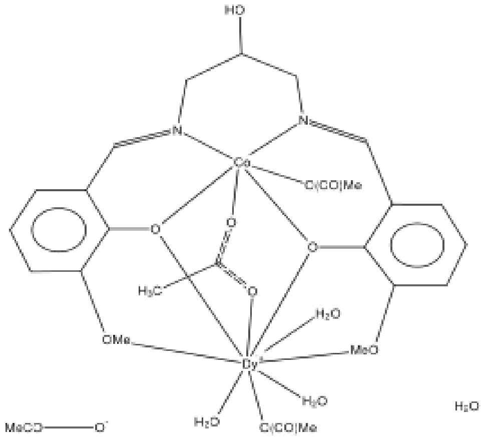	YUFKUH	2	CoDy	Yes	[7]
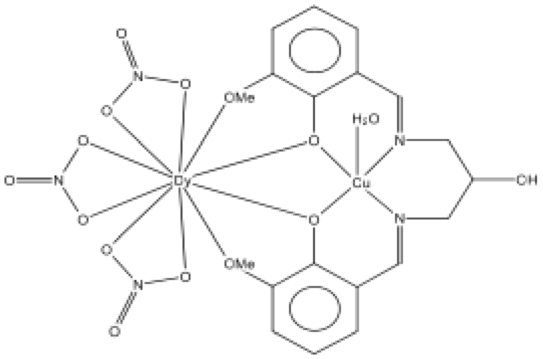	YUFWIH	2	CuDy	No	[7]

Structure	Refcode	Nuclearity	Metals	OH group[1]	Ref.
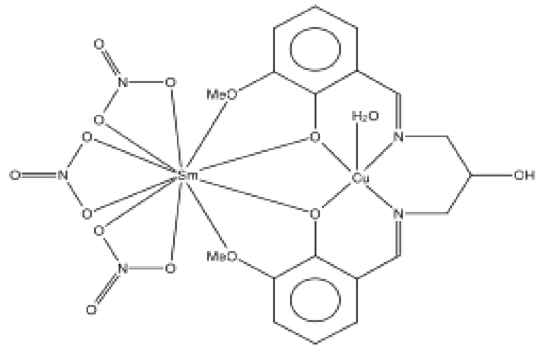	KEZYIZ	2	CuSm	No	[8]
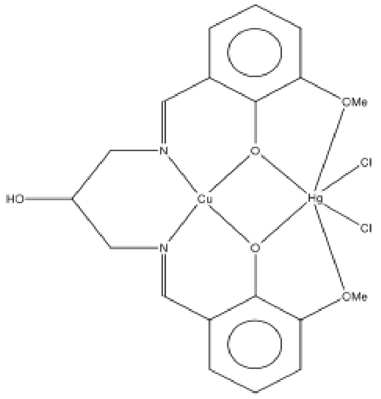	KEZYOF	2	CuHg	No	[8]
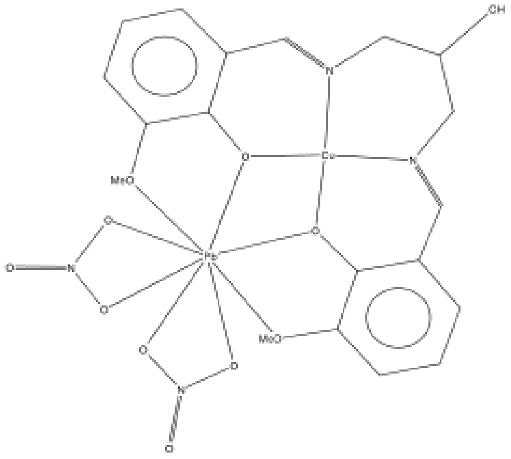	KEZYUL	2	CuPb	No	[8]
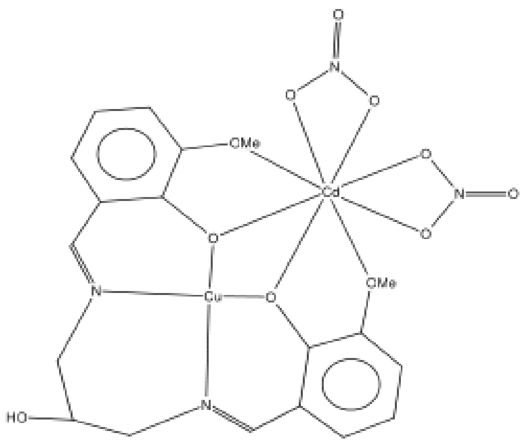	KEZZAS	2	CuCd	No	[8]

Structure	Refcode	Nuclearity	Metals	OH group[1]	Ref.
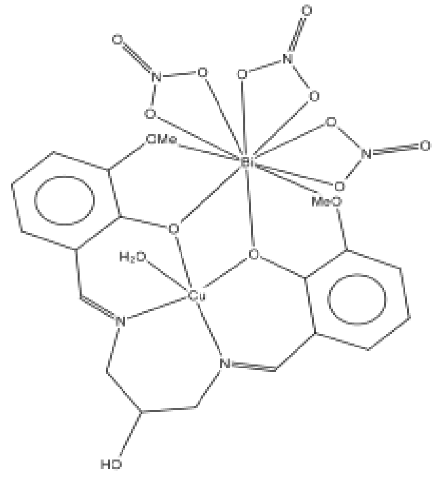	KEZZEW	2	CuBi	No	[8]
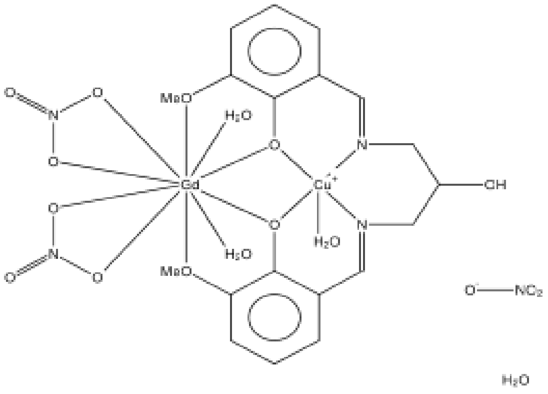	LUXBOX	2	CuGd	No	[9]
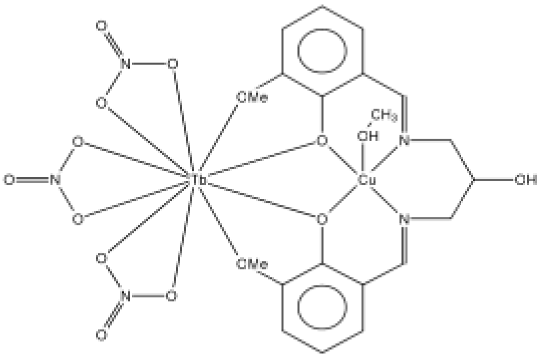	LUXBUD	2	CuTb	No	[9]
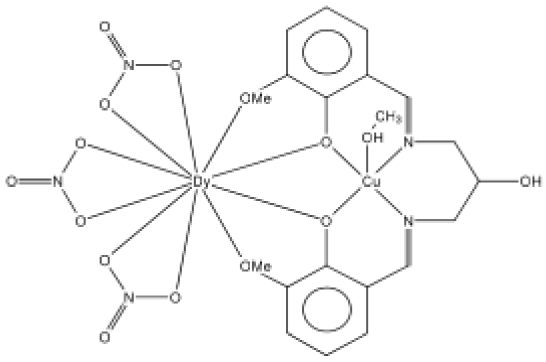	LUXCAK	2	CuDy	No	[9]

Structure	Refcode	Nuclearity	Metals	OH group[1]	Ref.
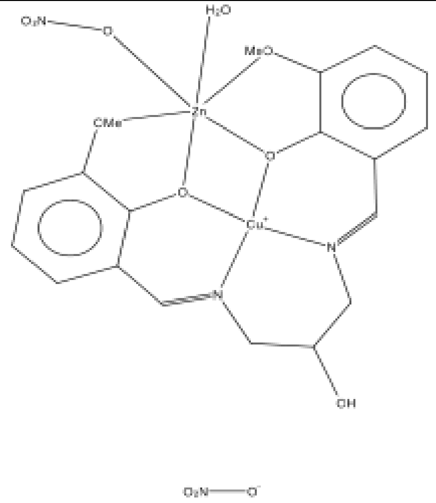	VEYLES	2	CuZn	No	[8]
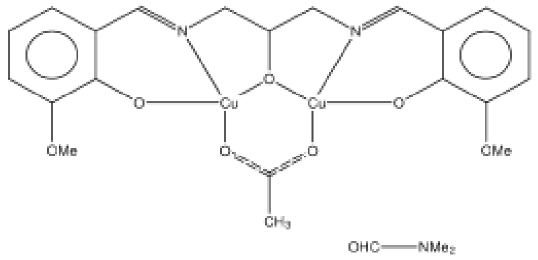	IWIHEB	2	Cu_2	Yes	[10]
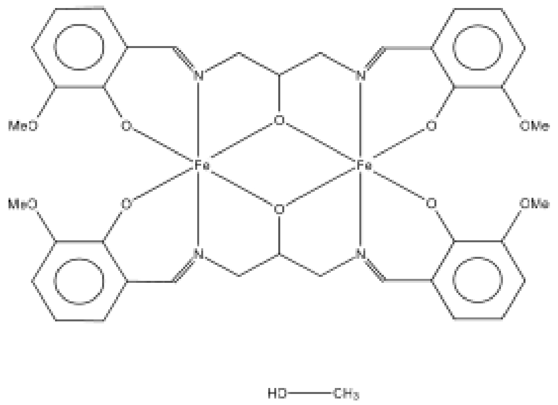	BOQLOJ	2	Fe_2	Yes	[11]
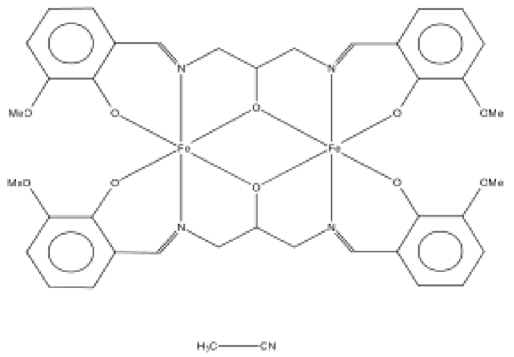	RINXUJ	2	Fe_2	Yes	[12]

Structure	Refcode	Nuclearity	Metals	OH group[1]	Ref.
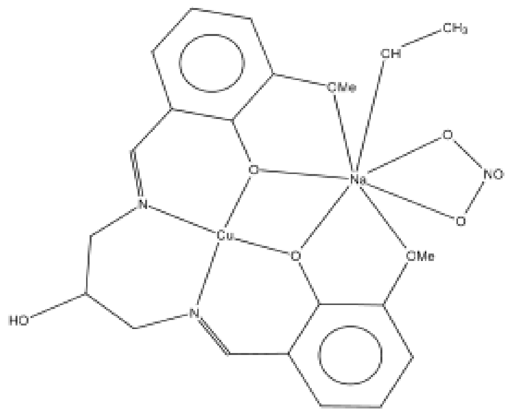	VEYLAO	2	CuNa	No	[8]
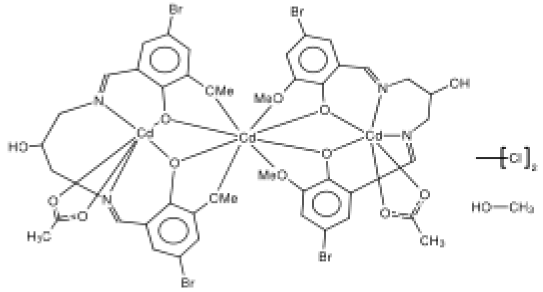	ENICAH	3	Cd_3	No	[13]
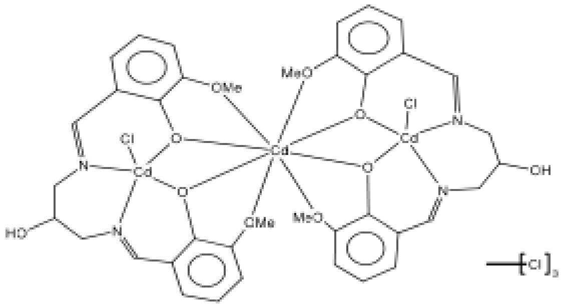	ENICEL	3	Cd_3	No	[13]
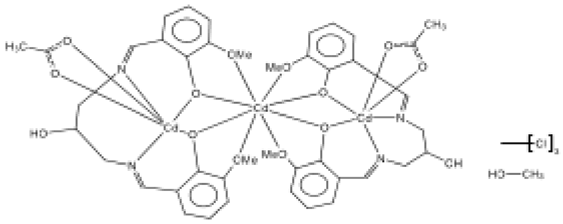	ENICIP	3	Cd_3	No	[13]
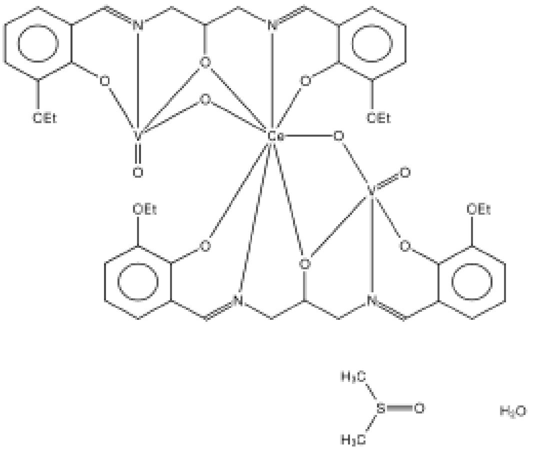	KEPQIG	3	V_2Ce	Yes	[14]

Structure	Refcode	Nuclearity	Metals	OH group[1]	Ref.
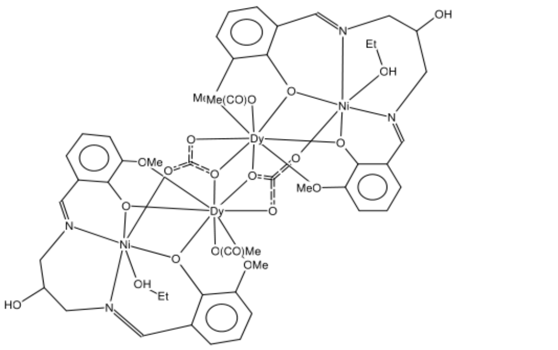	JASTUV01	4	Ni_2Dy_2	No	[5]
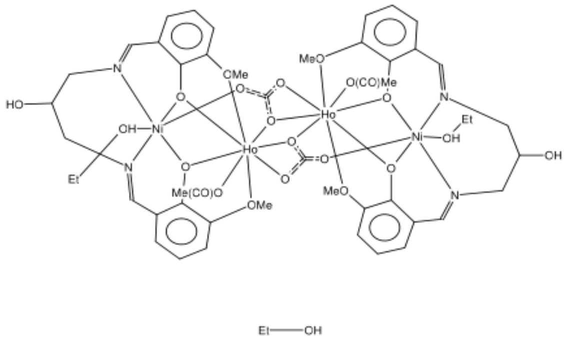	YECJUO	4	Ni_2Ho_2	No	[5]
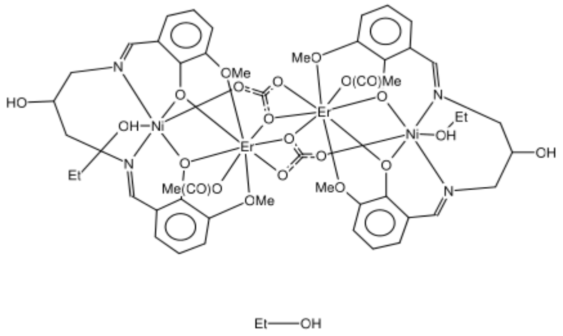	YECKAV	4	Ni_2Er_2	No	[5]
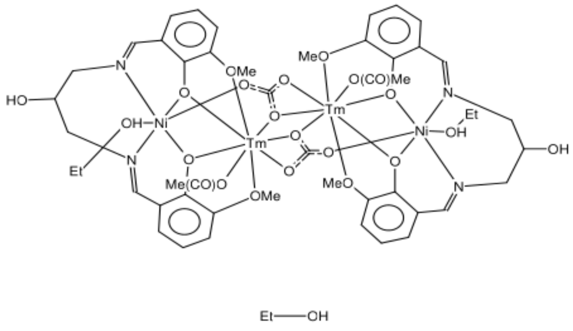	YECKEZ	4	Ni_2Tm_2	No	[5]

Structure	Refcode	Nuclearity	Metals	OH group[1]	Ref.
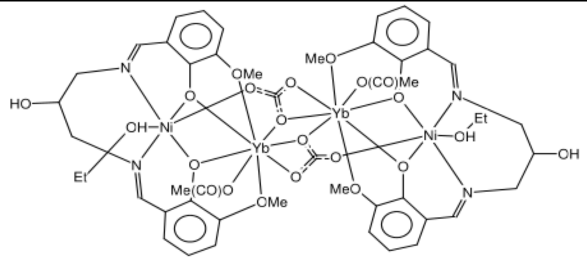	YECKID	4	Ni_2Yb_2	No	[5]
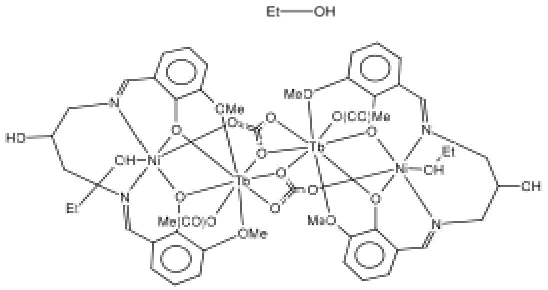	YECLOK	4	Ni_2Tb_2	No	[5]
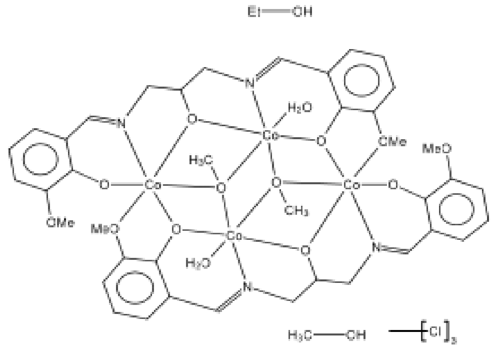	ENEZEE	4	Co_4	Yes	[13]
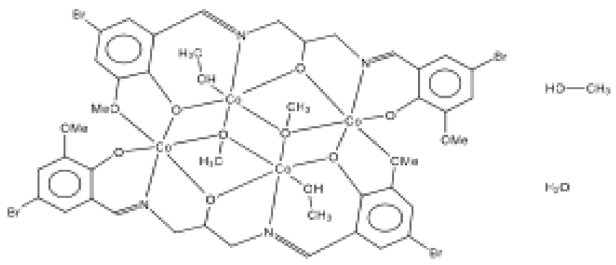	ENEZOO	4	Co_4	Yes	[13]
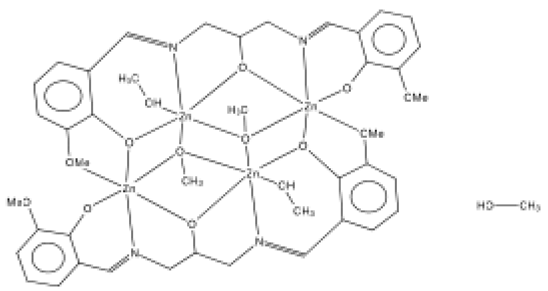	ENEZUU	4	Zn_4	Yes	[13]

Structure	Refcode	Nuclearity	Metals	OH group[1]	Ref.
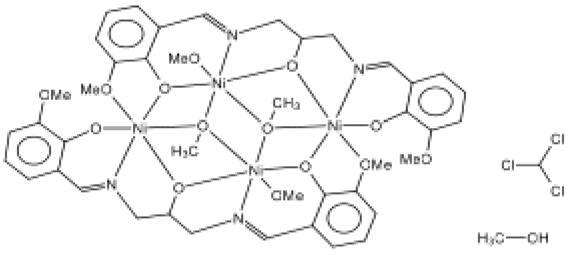	ENEZII	4	Ni_4	Yes	[13]
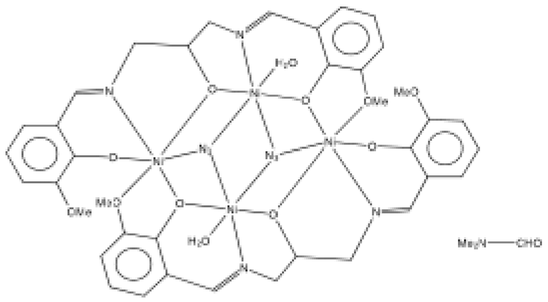	ENICOV	4	Ni_4	Yes	[13]
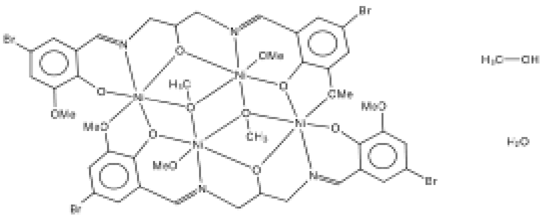	ENICUB	4	Ni_4	Yes	[13]
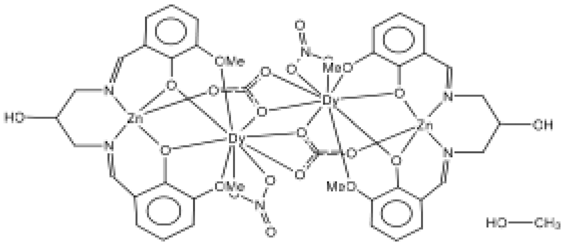	XASDAZ	4	Zn_2Dy_2	No	[15]
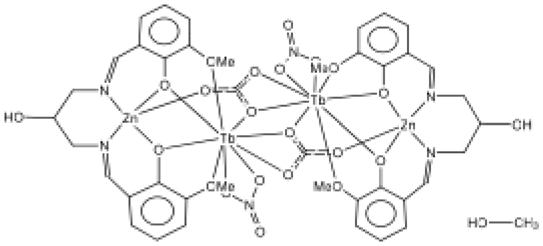	XASDED	4	Zn_2Tb_2	No	[15]
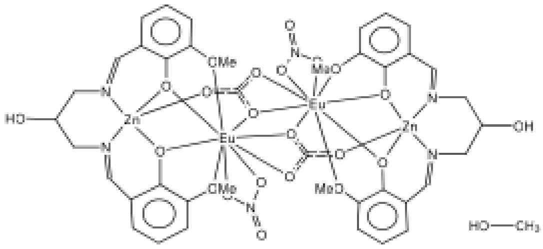	XASDIH	4	Zn_2Eu_2	No	[15]

Structure	Refcode	Nuclearity	Metals	OH group1	Ref.
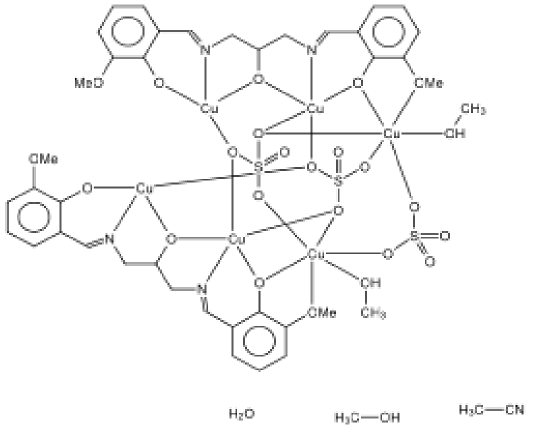	BOQMEA	6	Cu_6	Yes	[11]
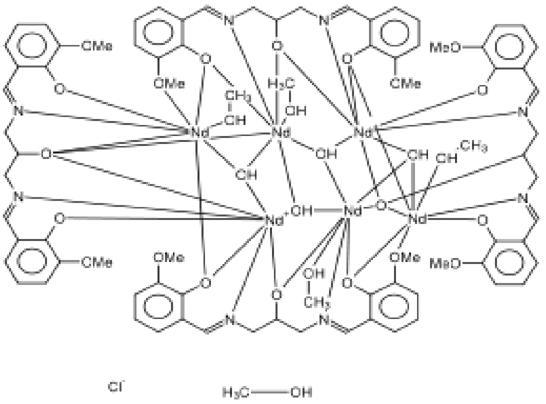	DAVZOR	6	Nd_6	Yes	[16]
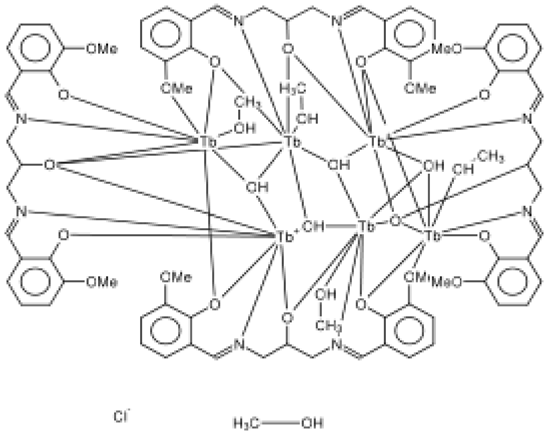	DAVZUX	6	Tb_6	Yes	[16]

Structure	Refcode	Nuclearity	Metals	OH group[1]	Ref.
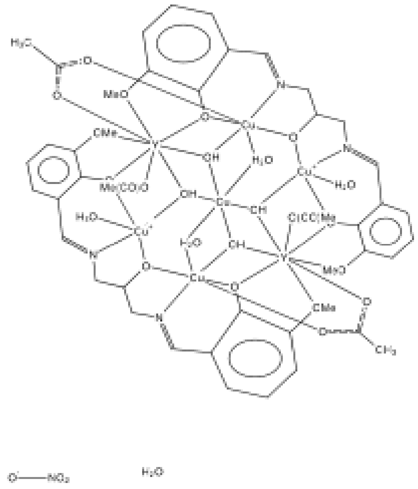	CONZAI	7	Cu_5V_2	Yes	[17]
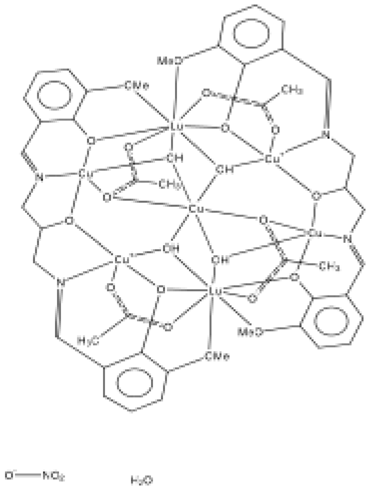	CONZEM	7	Cu_5Lu_2	Yes	[17]
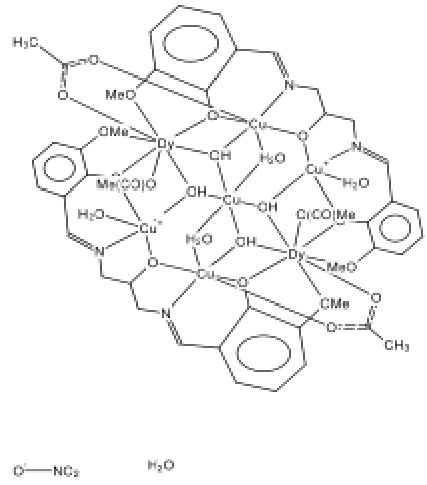	CONZIQ	7	Cu_5Dy_2	Yes	[17]

Structure	Refcode	Nuclearity	Metals	OH group[1]	Ref.
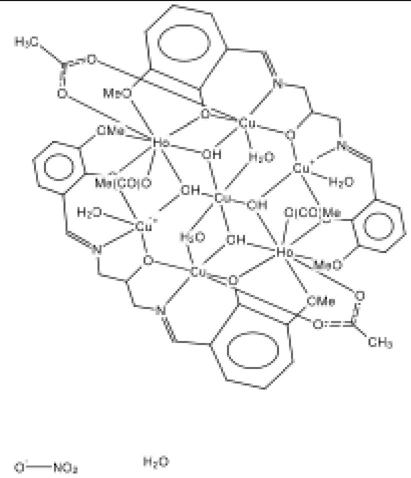	CONZOW	7	Cu_5Ho_2	Yes	[17]
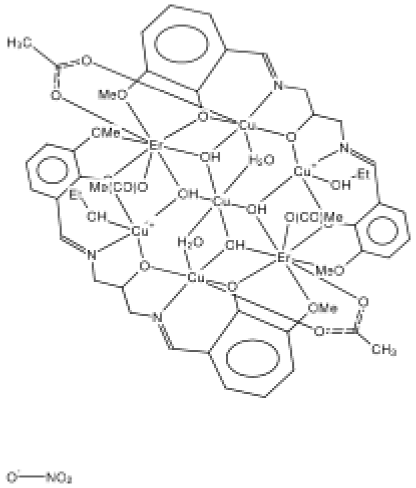	CONZUC	7	Cu_5Er_2	Yes	[17]
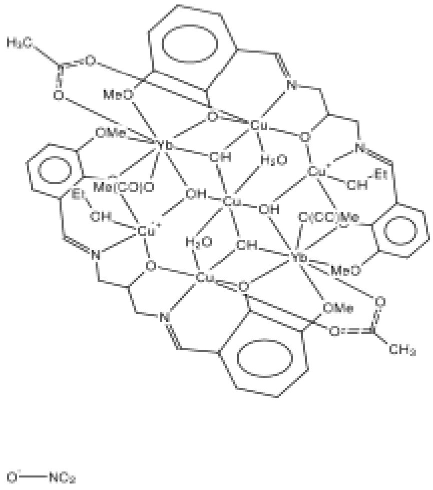	COPBAM	7	Cu_5Yb_2	Yes	[17]

Structure	Refcode	Nuclearity	Metals	OH group[1]	Ref.
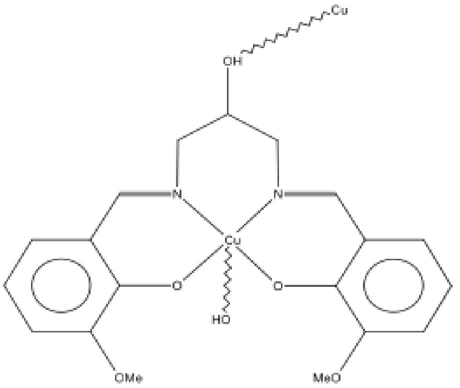	REFJIX	Polymer	{Cu}n	Yes	[18]
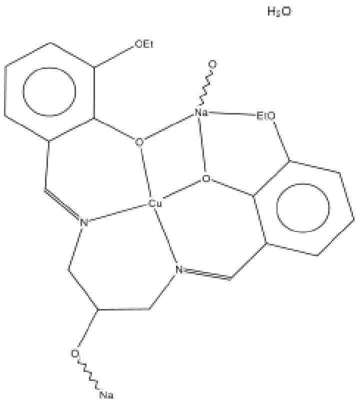	CIKPAP	polymer	{CuNa}n	Yes	[19]

References:

1. Mahboubi-Anarjan, P.; Bikas, R.; Hosseini-Monfared, H.; Aleshkevych, P.; Mayer, P. Synthesis, Characterization, EPR Spectroscopy and Catalytic Activity of a New oxidovanadium(IV) Complex with N2O2-Donor Ligand. *J. Mol. Struct.* **2017**, *1131*, 258–265; DOI:10.1016/J.MOLSTRUC.2016.11.059.

2. Smith, K. I.; Borer, L. L.; Olmstead, M. M. Vanadium(IV) and Vanadium(V) Complexes of Salicyladimine Ligands. *Inorg. Chem.* **2003**, *42* (23), 7410–7415; DOI:10.1021/ic034640p.

3. Tsuchimoto, M.; Ishii, T.; Imaoka, T.; Yamamoto, K. Synthesis and Electrochemical Properties of Oxovanadium Complexes with a Pentadentate Schiff Base Ligand. *Bull. Chem. Soc. Jpn.* **2004**, *77* (10), 1849–1854; DOI:10.1246/bcsj.77.1849.

4. Datta, A.; Das, K.; Massera, C.; Clegg, J. K.; Sinha, C.; Huang, J.-H.; Garribba, E. A Mixed Valent Heterometallic Cu ^{II} /Na ^{I} Coordination Polymer with Sodium–phenyl Bonds. *Dalt. Trans.* **2014**, *43* (14), 5558–5563; DOI:10.1039/C4DT00189C.

5. Jiang, L.; Liu, Y.; Liu, X.; Tian, J.; Yan, S. Three Series of Heterometallic Ni ^{II} –Ln ^{III} Schiff Base Complexes: Synthesis, Crystal Structures and Magnetic Characterization. *Dalt. Trans.* **2017**, *46* (37), 12558–12573; DOI:10.1039/C7DT02351K.

6. Jiang, L. *CSD Communication, Private Communication*; 2017.

7. Dolai, M.; Ali, M.; Titiš, J.; Boča, R. Cu(Ii)–Dy(Iii) and Co(Iii)–Dy(Iii) Based Single Molecule Magnets with Multiple Slow Magnetic Relaxation Processes in the Cu(Ii)–Dy(Iii) Complex. *Dalt. Trans.* **2015**, *44* (29), 13242–13249; DOI:10.1039/C5DT00960J.

8. Dolai, M.; Mistri, T.; Panja, A.; Ali, M. Diversity in Supramolecular Self-Assembly through Hydrogen-Bonding Interactions of Non-Coordinated Aliphatic –OH Group in a Series of Heterodinuclear CuIIM

(M = NaI, ZnII, HgII, SmIII, BiIII, PbII and CdII). *Inorganica Chim. Acta* **2013**, *399*, 95–104; DOI:10.1016/J.ICA.2013.01.006.

9. Chiboub Fellah, F. Z.; Boulefred, S.; Chiboub Fellah, A.; El Rez, B.; Duhayon, C.; Sutter, J.-P. Binuclear CuLn Complexes (LnIII = Gd, Tb, Dy) of Alcohol-Functionalized Bicompartmental Schiff-Base Ligand. Hydrogen Bonding and Magnetic Behaviors. *Inorganica Chim. Acta* **2016**, *439*, 24–29; DOI:10.1016/J.ICA.2015.09.032.

10. Elmali, A.; Zeyrek, C. T.; Elerman, Y. Crystal Structure, Magnetic Properties and Molecular Orbital Calculations of a Binuclear copper(II) Complex Bridged by an Alkoxo-Oxygen Atom and an Acetate Ion. *J. Mol. Struct.* **2004**, *693* (1–3), 225–234; DOI:10.1016/J.MOLSTRUC.2004.02.037.

11. Lan, Y.; Novitchi, G.; Clérac, R.; Tang, J.-K.; Madhu, N. T.; Hewitt, I. J.; Anson, C. E.; Brooker, S.; Powell, A. K. Di-, Tetra- and Hexanuclear iron(III), manganese(II/III) and copper(II) Complexes of Schiff-Base Ligands Derived from 6-Substituted-2-Formylphenols. *Dalt. Trans.* **2009**, *10*, 1721; DOI:10.1039/b818113f.

12. Mitra, M.; Maji, A. K.; Ghosh, B. K.; Raghavaiah, P.; Ribas, J.; Ghosh, R. Catecholase Activity of a Structurally Characterized Dinuclear iron(III) Complex [FeIII2(L)2] [H3L = N,N′-bis(3-Methoxysalicylaldimine)-1,3-Diaminopropan-2-Ol]. *Polyhedron* **2014**, *67*, 19–26; DOI:10.1016/J.POLY.2013.08.064.

13. Jiang, L.; Zhang, D.-Y.; Suo, J.-J.; Gu, W.; Tian, J.-L.; Liu, X.; Yan, S.-P. Synthesis, Magnetism and Spectral Studies of Six Defective Dicubane Tetranuclear {M_4O_6} (M = Ni^{II}, Co^{II}, Zn^{II}) and Three Trinuclear Cd^{II} Complexes with Polydentate Schiff Base Ligands. *Dalt. Trans.* **2016**, *45* (25), 10233–10248; DOI:10.1039/C6DT00380J.

14. Tsuchimoto, M.; Ishii, T.; Imaoka, T.; Yamamoto, K.; Yoshioka, N.; Sunatsuki, Y. Synthesis and Structures of Vanadium–Cerium Trinuclear Complexes with Schiff-Base Ligands. *Bull. Chem. Soc. Jpn.* **2006**, *79* (9), 1393–1397; DOI:10.1246/bcsj.79.1393.

15. Griffiths, K.; Mayans, J.; Shipman, M. A.; Tizzard, G. J.; Coles, S. J.; Blight, B. A.; Escuer, A.; Kostakis, G. E. Four New Families of Polynuclear Zn-Ln Coordination Clusters. Synthetic, Topological, Magnetic, and Luminescent Aspects. *Cryst. Growth Des.* **2017**, *17* (4), 1524–1538; DOI:10.1021/acs.cgd.6b01401.

16. Liao, S.; Yang, X.; Jones, R. A. Self-Assembly of Luminescent Hexanuclear Lanthanide Salen Complexes. *Cryst. Growth Des.* **2012**, *12* (2), 970–974; DOI:10.1021/cg201444p.

17. Chandrasekhar, V.; Dey, A.; Das, S.; Rouzières, M.; Clérac, R. Syntheses, Structures, and Magnetic Properties of a Family of Heterometallic Heptanuclear [Cu_5Ln_2] (Ln = Y(III), Lu(III), Dy(III), Ho(III), Er(III), and Yb(III)) Complexes: Observation of SMM Behavior for the Dy(III) and Ho(III) Analogues. *Inorg. Chem.* **2013**, *52* (5), 2588–2598; DOI:10.1021/ic302614k.

18. Datta, A.; Clegg, J. K.; Huang, J.-H.; Pevec, A.; Garribba, E.; Fondo, M. Hydroxo-Bridged 1-D Coordination Polymer of Cu(II) Incorporating with Salicyladimine Precursor: Spectral and Temperature Dependent Magneto Structural Correlation. *Inorg. Chem. Commun.* **2012**, *24*, 216–220; DOI:10.1016/J.INOCHE.2012.07.017.

19. Biswas, D.; Chakrabarty, P. P.; Saha, S.; Jana, A. D.; Schollmeyer, D.; García-Granda, S. Ligand Mediated Structural Diversity and Role of Different Weak Interactions in Molecular Self-Assembly of a Series of copper(II)–sodium(I) Schiff-Base Heterometallic Complexes. *Inorganica Chim. Acta* **2013**, *408*, 172–180; DOI:10.1016/J.ICA.2013.09.011.

Table S2. Crystallographic data for complexes **1-5**.

**Identification code**	**1**	**2**	**3**	**4**	**5**
Empirical formula	$C_{42}H_{48}N_5O_{20}Br_4Zn_2Nd$	$C_{45}H_{54.67}N_5O_{20.33}Br_4Zn_2Sm$	$C_{42}H_{52.67}N_5O_{22.33}Br_4Zn_2Eu$	$C_{42}H_{52.67}N_5O_{22.33}Br_4Zn_2Tb$	$C_{42}H_{52.67}N_5O_{22.33}$ Br_4Zn_2Dy
Formula weight	1537.47	1591.07	1587.23	1594.19	1597.77
Temperature/K	293.0	120(1)	120(1)	293.0	120(1)
Crystal system	monoclinic	monoclinic	trigonal	trigonal	trigonal
Space group	$P2_1/n$	$P2_1/n$	R-3c	R-3c	R-3c
a/Å	9.9805(4)	10.2675(2)	19.8962(4)	20.0572(5)	19.8706(5)
b/Å	24.132(1)	23.8581(3)	19.8962(4)	20.0572(5)	19.8706(5)
c/Å	22.9091(7)	22.5353(3)	76.655(2)	76.388(3)	76.007(2)
α/°	90	90	90	90	90
β/°	91.55(1)	91.261(1)	90	90	90
γ/°	90	90	120	120	120
Volume/Å^3	5515.4(4)	5518.9(1)	26279.2(9)	26613(1)	25990(1)
Z	4	4	18	18	18
ρ_{calc}/ g/cm^3	1.852	1.915	1.7976	1.790	1.838
μ/mm^{-1}	4.763	12.967	12.414	4.765	11.701
F(000)	3020	3136.0	13720.0	14100.0	14118.0
Crystal size/mm^3	0.25 × 0.12 × 0.02	0.4 × 0.35 × 0.02	0.47 × 0.29 × 0.07	0.30 × 0.30 × 0.20	0.59 × 0.34 × 0.08
Radiation	Mo Kα (λ = 0.71073)	Cu Kα (λ = 1.54184)	Cu Kα (λ = 1.54184)	Mo Kα (λ = 0.71073)	Cu Kα (λ = 1.54184)
2Θ range /°	5.3 to 50.48	7.42 to 152.88	8.88 to 135.34	4.8 to 50.48	8.9 to 135.36
Reflections collected	41616	38779	58409	70659	35163
Independent reflections	9953 [R_{int} = 0.0806, R_{sigma} = 0.0852]	11370 [R_{int} = 0.0548, R_{sigma} = 0.0444]	5291 [R_{int} = 0.1573, R_{sigma} = 0.0539]	5349 [R_{int} = 0.0860, R_{sigma} = 0.0376]	5229 [R_{int} = 0.0842, R_{sigma} = 0.0405]
Data/restr./parameters	9953/0/659	11370/0/699	5291/6/350	5349/6/352	5229/6/350
Goodness-of-fit on F^2	1.042	1.083	1.034	1.107	1.039
Final R indexes [I>=2σ (I)]	R_1 = 0.0593, wR_2 = 0.1250	R_1 = 0.0481, wR_2 = 0.1314	R_1 = 0.0785, wR_2 = 0.2180	R_1 = 0.0561, wR_2 = 0.1505	R_1 = 0.0610, wR_2 = 0.1621
Final R indexes [all data]	R_1 = 0.1121, wR_2 = 0.1544	R_1 = 0.0540, wR_2 = 0.1384	R_1 = 0.0817, wR_2 = 0.2254	R_1 = 0.0789, wR_2 = 0.1704	R_1 = 0.0661, wR_2 = 0.1699
Larg. diff. peak/hole / e Å^{-3}	2.21/-1.72	2.29/-1.59	2.76/-1.73	2.08/-0.82	2.07/-1.24
CCDC No.	1849667	1849666	1849668	1849669	1849665

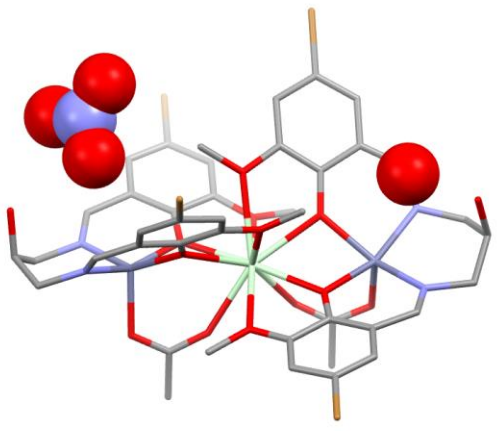

Figure S1. Location of hydrogen bonded NO_3^- anion and water molecule in pockets formed by 2-hydroxypropyl groups in complex $Zn^{II}–Nd^{III}–Zn^{II}$ (**1**).

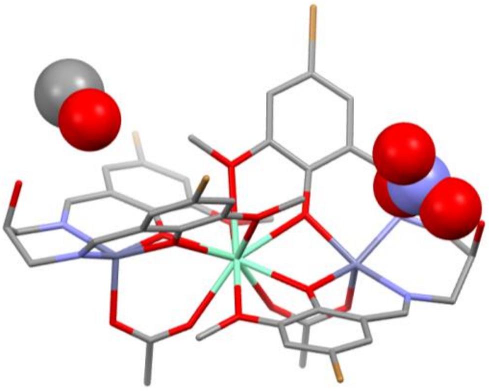

Figure S2. Location of hydrogen bonded NO_3^- anion and methanol molecule in pockets formed by 2-hydroxypropyl groups in complex $Zn^{II}–Sm^{III}–Zn^{II}$ (**2**).

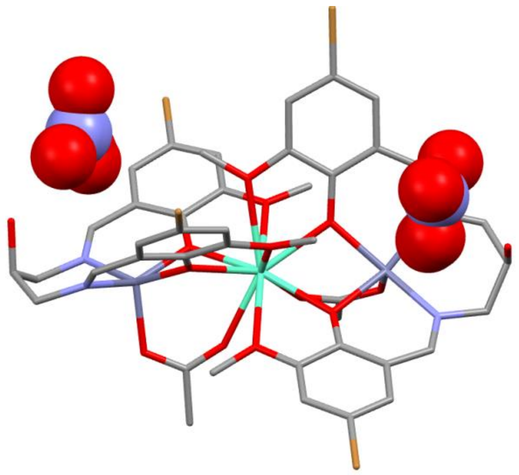

Figure S3. Location of disordered hydrogen bonded NO_3^- anion and water molecule in pockets formed by 2-hydroxypropyl groups in complex $Zn^{II}–Eu^{III}–Zn^{II}$ (**3**).

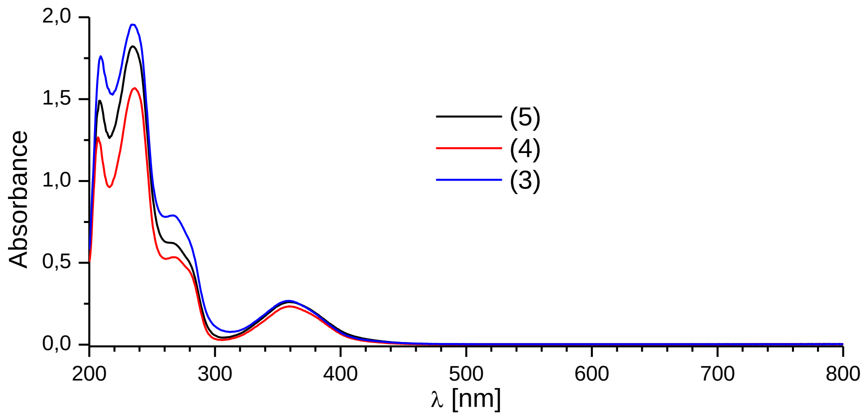

Figure S4. The absorbance spectra of compounds Zn^{II}–Eu^{III}–Zn^{II} (**3**), Zn^{II}–Tb^{III}–Zn^{II} (**4**) and Zn^{II}–Dy^{III}–Zn^{II} (**5**) in methanol solution (~$2 \cdot 10^{-5}$ M).

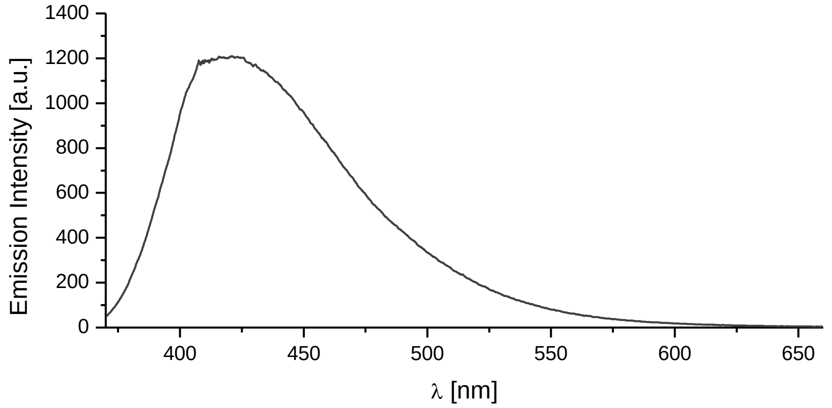

Figure S5. Luminescence spectrum of complex **1** (Zn^{II}–Nd^{III}–Zn^{II}) in methanol solution (~$2 \cdot 10^{-5}$ M).

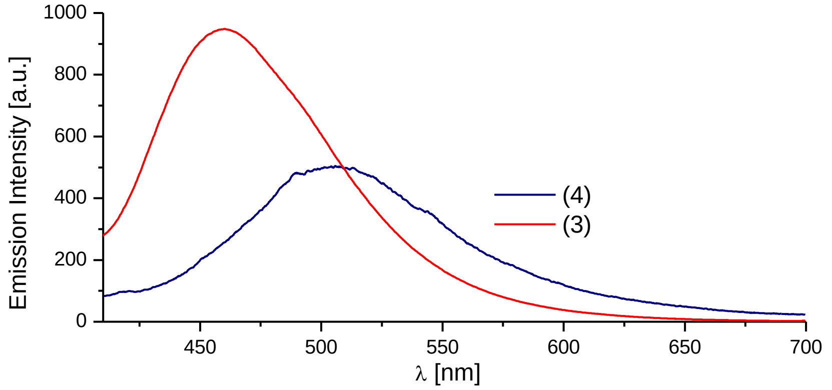

Figure S6. Solid state emission spectra of complexes **3** (Zn^{II}–Eu^{III}–Zn^{II}) and **4** (Zn^{II}–Tb^{III}–Zn^{II}).

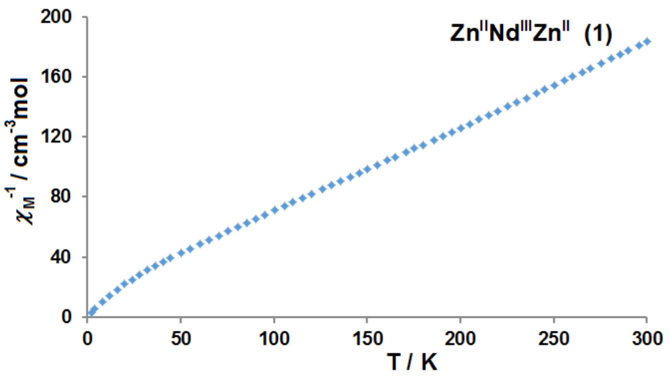

Figure S7. Temperature dependence of the experimental χ_M^{-1} versus T for $Zn^{II}–Nd^{III}–Zn^{II}$ (**1**).

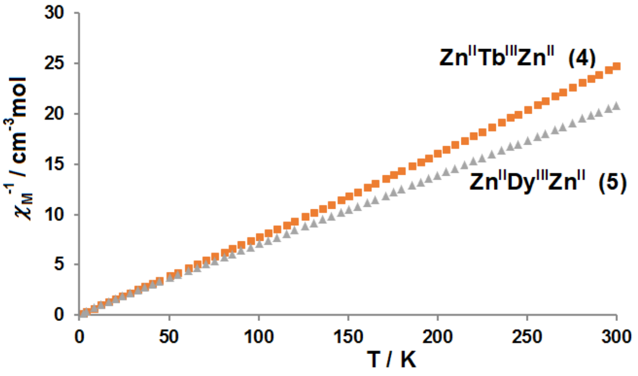

Figure S8. Temperature dependence of the experimental χ_M^{-1} versus T for $Zn^{II}–Tb^{III}–Zn^{II}$ (**4**) and $Zn^{II}–Dy^{III}–Zn^{II}$ (**5**).

Table S3. Theoretical and experimental values of the $\chi_M T$ product for compounds **1**, **4**, **5** at the room temperature.

**Complex**	**No. of *f* electrons**	**Ground state free ion symbol ($^{2S+1}L_J$)**	**g_{Ln}**	**$\chi_M T_{theor}$/ cm^{3}mol^{-1}K (Ln^{III})**	**$\chi_M T_{exp}$/ cm^{3}mol^{-1}K ($Zn^{II}_2Ln^{III}$)**
**1** ($Zn^{II}–Nd^{III}–Zn^{II}$)	3	$^4I_{9/2}$	8/11	1.64	1.62
**4** ($Zn^{II}–Tb^{III}–Zn^{II}$)	8	$^6F_6$	3/2	11.82	12.10
**5** ($Zn^{II}–Dy^{III}–Zn^{II}$)	9	$^4H_{15/2}$	4/3	14.17	14.39

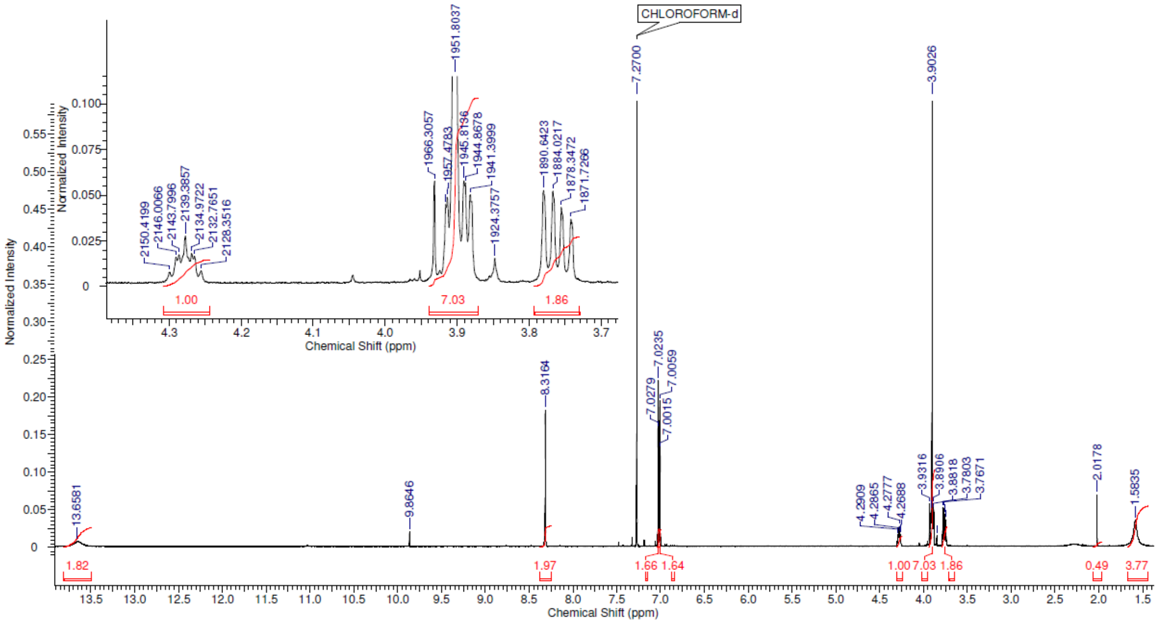

Figure S9. ^{1}H NMR spectrum of ligand **H_3L** in $CDCl_3$ solution.

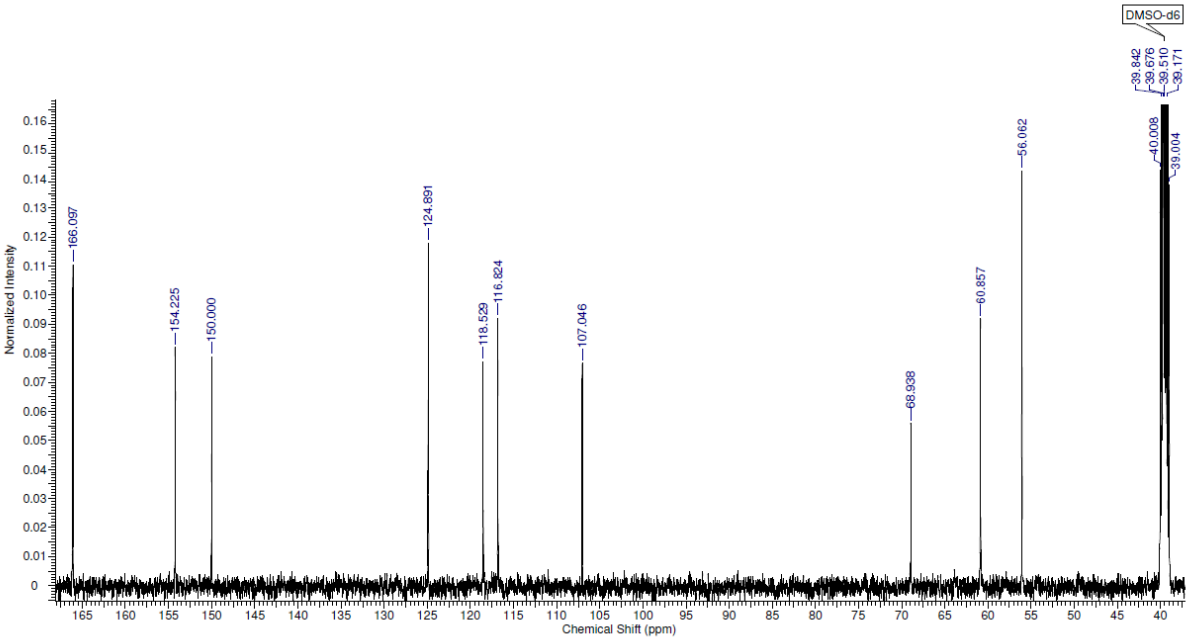

Figure S10. ^{13}C NMR spectrum of ligand **H_3L** in DMSO-d_6 solution.
